# Evaluating the impact of doxorubicin preconditioning on the efficacy of inhaled recombinant human IL-15 immunotherapy in dogs with pulmonary metastasis

**DOI:** 10.1186/s44356-025-00040-5

**Published:** 2025-10-01

**Authors:** Madison E. Luker, Daniel York, Sami Al-Nadaf, Eric G. Johnson, Sita S. Withers, Sylvia M. Cruz, Amandine Lejeune, Katherine A. Skorupski, Jacque Young, Tamar Cohen-Davidyan, Ellen E. Sparger, William TN Culp, William J. Murphy, Michael S. Kent, Robert J. Canter, Robert B. Rebhun

**Affiliations:** 1 Department of Surgical and Radiological Sciences, School of Veterinary Medicine, University of California Davis, Davis, CA USA; 2 School of Animal and Veterinary Sciences, University of Adelaide, Roseworthy, South Australia Australia; 3 Department of Surgery, Division of Surgical Oncology, School of Medicine, University of California Davis, Sacramento, CA USA; 4 William R. Pritchard Veterinary Medical Teaching Hospital, School of Veterinary Medicine, University of California Davis, Davis, CA USA; 5 Department of Medicine and Epidemiology, School of Veterinary Medicine, University of California Davis, Davis, CA 95616 USA; 6 Department of Dermatology, School of Medicine, University of California Davis, Sacramento, CA USA; 7 Department of Internal Medicine, Division of Hematology and Oncology, School of Medicine, University of California Davis, Sacramento, CA USA

**Keywords:** Inhaled IL-15, Immunotherapy, Doxorubicin, Canine cancer, Osteosarcoma, Melanoma, Lung metastasis

## Abstract

**Background:**

Canine osteosarcoma (OSA) and melanoma are highly aggressive cancers with a high propensity for lung metastasis. A recent phase 1 trial demonstrated that inhaled recombinant human interleukin-15 (rhIL-15) immunotherapy is safe and may be effective against lung metastases in dogs with metastatic OSA or melanoma. Notably, baseline lymphopenia correlated with clinical benefit in that trial. Building on these findings, this study evaluates whether preconditioning with doxorubicin to induce transient lymphodepletion before inhaled rhIL-15 immunotherapy improves the overall response rate in dogs with metastatic melanoma or tumors of bone.

**Methods:**

Dogs with established pulmonary metastases from melanoma or bone tumors were treated with doxorubicin (30 mg/m^2^ or 1 mg/kg) 7 days before starting inhaled rhIL-15 (50 µg twice daily x 14 days). Response rate was the primary objective of this study. Secondary objectives included toxicity assessment, immune correlative analyses via hematological monitoring, and NanoString transcriptomics analysis.

**Results:**

Ten dogs were enrolled, with eight reaching the Day 35 evaluable response assessment. One dog achieved a complete response lasting over 1 year, and two had stable disease, yielding an overall clinical benefit rate (CBR) of 30% and an overall response rate (ORR) of 10%. Doxorubicin preconditioning did not alter the toxicity of inhaled rhIL-15 therapy. While doxorubicin treatment decreased absolute lymphocyte counts (ALC), as expected, this reduction did not improve the CBR or ORR compared to the prior study evaluating inhaled rhIL-15 monotherapy. Transcriptomic analysis of peripheral blood mononuclear cells did not reveal a significant increase in natural killer or cytotoxic T-cell population frequencies following doxorubicin and inhaled rhIL-15 therapy.

**Conclusions:**

Preconditioning with standard doses of doxorubicin does not alter the clinical response or safety profile of inhaled rhIL-15 therapy in dogs with advanced pulmonary metastatic disease. Further research is needed to evaluate combinatorial treatment strategies that enhance the efficacy of inhaled rhIL-15 in dogs with advanced metastatic disease.

**Supplementary Information:**

The online version contains supplementary material available at 10.1186/s44356-025-00040-5.

## Background

Dogs with spontaneous cancers serve as a valuable translational model for evaluating novel immunotherapy approaches. Specifically, naturally occurring canine osteosarcoma (OSA) and melanoma provide an important bridge between preclinical mouse studies and human clinical trials [1, 2]. These cancers arise in dogs with a fully intact immune system, share similar genetic and molecular aberrations to human OSA and melanoma, and follow a comparable but accelerated clinical disease progression to their human counterparts [2–7]. One promising treatment modality for dogs with metastatic OSA or melanoma is inhaled recombinant cytokine therapy using human interleukin-2 (IL-2) or interleukin-15 (rhIL-15) [8, 9]. Previously, it has been demonstrated that inhaled liposomal and free human IL-2 reaches the lungs of nebulized dogs and remains in the lungs for at least 22 h [10]. Additionally, dogs treated with inhaled rhIL-15 have detectable circulating rhIL-15 in their plasma [8]. IL-15 enhances the immune response by stimulating CD8 + memory T lymphocytes and natural killer (NK) cells, making it an attractive therapeutic option for immunosuppressive cancers like OSA [6, 11]. Inhaled cytokine therapy provides the distinct advantage of directly targeting the lungs, the most common and deleterious site of OSA and melanoma metastasis. This regional delivery also limits systemic exposure and toxicity, a frequent limitation of intravenous cytokine therapy [8, 11].

A recent phase 1 trial evaluating inhaled rhIL-15 in dogs with metastatic OSA and melanoma demonstrated that the therapy was well tolerated and induced durable responses in a subset of dogs. Furthermore, dogs with a lower baseline absolute lymphocyte count (ALC) were statistically more likely to experience clinical benefit and showed a greater fold increase in ALC after treatment. Therefore, we hypothesized that reduced systemic lymphocyte populations might decrease ‘cytokine sinks’ [12], improving NK and CD8 + T cell access to rhIL-15 and thereby enabling them to expand more effectively and enhance the clinical benefit following treatment. Lymphodepletion and its corresponding outcomes—improving availability of cytokines and creating space to enhance the expansion and survival of desired immune cell populations– is regularly utilized and is known to improve the efficacy of adoptive cell transfer [13]. One common approach for inducing lymphodepletion in human patients is chemotherapy [14]. In tumor-bearing dogs, the chemotherapeutic agent doxorubicin has been shown to reduce circulating T lymphocytes without selectivity for T regulatory cells [11]. Given our previous findings, we were interested in whether preconditioning with doxorubicin to induce transient lympho-reduction before inhaled rhIL-15 immunotherapy would improve the overall response rate (ORR) compared to inhaled rhIL-15 alone. Therefore, we assessed whether preconditioning with doxorubicin was safe and enhanced the efficacy of inhaled rhIL-15 in dogs with metastatic OSA or melanoma. Additionally, our objective was to further characterize the immunologic effects of doxorubicin and inhaled rhIL-15 to identify shifts in peripheral blood mononuclear cells (PBMC) gene expression and immune cell populations.

## Methods

### Patient enrollment

Complete patient eligibility is outlined within the study operations manual (SOM) (supplemental information). Briefly, this trial recruited client-owned dogs with naturally occurring metastatic bone tumors or melanoma at the UC Davis Veterinary Medical Teaching Hospital (VMTH). Dogs were required to be over 1 year old, weigh at least 5 kg, have a Veterinary Cooperative Oncology Group-Common Terminology Criteria for Adverse Events (VCOG-CTCAE v2) performance score less than 2, and present with one or more lung lesions measuring at least 1 cm in diameter on thoracic radiographs, consistent with gross pulmonary metastatic disease from histologically or cytologically confirmed melanoma or tumors of bone [15]. Multidrug resistance (MDR) mutation status was determined for at-risk breeds, and a positive mutation status excluded patient enrollment. Dogs were not eligible for enrollment if they had surgery or chemotherapy within 2 weeks of Day 0 or immunotherapy or radiation therapy within 4 weeks of Day 0. Dogs were also not eligible for enrollment if they had prior rhIL-15 therapy. Dogs with prior or suspected cardiac disease based on a screening echocardiogram were excluded from the trial. Concurrent therapies were not allowed, except non-steroidal anti-inflammatory drugs (NSAIDs) for pain management. All dogs were assessed for adequate organ function through a serum chemistry panel, urinalysis, and complete blood count (CBC). If classified as having inadequate organ function per the inclusion criteria, the dog was excluded from the trial (see supplemental SOM). Thoracic radiographs were performed within 7 days preceding Day 0. The trial was approved by the UC Davis VMTH Clinical Trial Review Board and the UC Davis IACUC (IACUC#22674). All owners signed informed owner consent forms.

### Study design and treatment

**Fig. 1:**
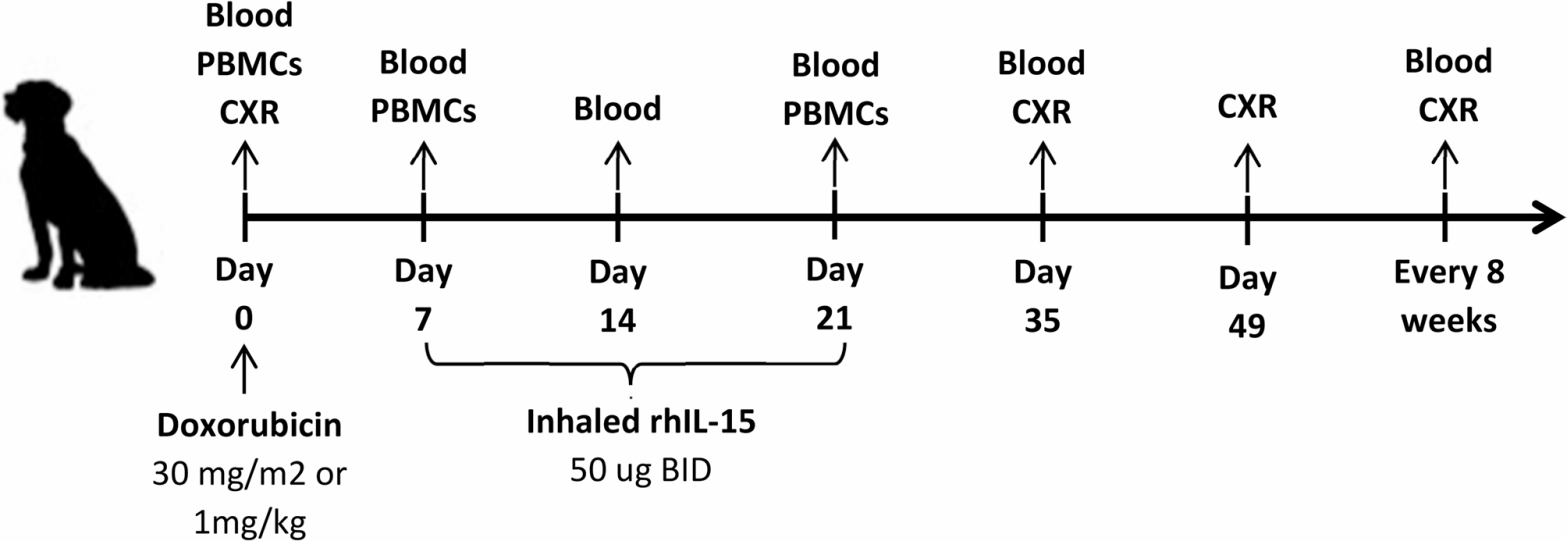
Study design of clinical trial. Schema of canine clinical trial of doxorubicin and inhaled rhIL-15. Doxorubicin was administered on Day 0, and inhaled rhIL-15 was delivered by nebulizer twice daily for 14 days, starting on Day 7. Blood was drawn prior to treatment and on days 0, 7, 14, 21, and 35. Peripheral blood mononuclear cells (PBMCs) were isolated on Days 0, 7, and 21. Response was evaluated by thoracic radiograph (CXR) on Days 0, 35, 49, and every 8 weeks thereafter

The trial schema is provided in Fig. 1. On Day 0, dogs received doxorubicin at the standard accepted clinical dose of 30 mg/m2 for dogs weighing more than 15 kg or 1 mg/kg for dogs weighing 15 kg or less [16]. Starting on Day 7, patients received inhaled rhIL-15 twice daily for 14 days at the Maximum tolerated dose level of 50 ug, as established in the previous phase 1 trial [8]. For each patient, the first inhaled rhIL-15 treatment on Day 7 was administered at the UC Davis Veterinary Medical Teaching Hospital (VMTH) to monitor patient tolerance. The subsequent treatments, for a total of 28, were administered at home by the owners, who received proper training on treatment administration. All treatments were administered at least 8 h apart. The nebulizer and protocol were as described in the previous trial [8].

### Clinical and investigational assessments

Clinical assessments, including physical exam and vital signs, were performed at all study visits. On Day 7, temperature and blood pressure were measured before the first inhaled rhIL-15 treatment and 1 h, 3 h, and 5 h post-treatment. Follow-up visits, including physical examinations, CBC, and PBMC collections, were performed before doxorubicin administration on Day 0, before initiation of inhaled rhIL-15 on Day 7, and on Day 14, and Day 21 (Fig. 1). Serum chemistry panels were performed prior to Day 0, on Day 7, and Day 21. Urinalyses were performed before enrollment and on Day 21. Toxicity was assessed using VCOG-CTCAE v.2 consensus document for adverse events (AEs) [15]. Response and progression were assessed by a board-certified veterinary radiologist (EJ) using response evaluation criteria for solid tumors in dogs (RECIST), with the exception that target lung metastatic lesions were required to have a minimum diameter of 1 cm instead of 2 cm, as described in our phase 1 trial [8, 17]. Three-view thoracic radiographs were performed within 7 days preceding Day 0 and repeated on Days 35, 49, and every 8 weeks after Day 49. A minimum duration of 28 days between imaging was required to be considered SD, PR, or CR. Pseudo-progression was defined as a PR or CR occurring after initial documentation of PD in the absence of other systemic treatment. The CBR was defined as the percentage of cases achieving SD, PR, and CR. OS was defined as the time from Day 0 to the day of death. Death was defined as either a naturally occurring death or euthanasia. PFS was defined as the time from Day 0 to progressive disease (new lung lesions identified and/or > 20% increase in the longest tumor diameter of target lesions).

### NanoString nCounter

Whole blood was collected in EDTA tubes from patients at Day 0 (pre-doxorubicin), Day 7 (post doxorubicin), and Day 21 (post-rhIL-15). PBMCs were isolated via Ficoll-Paque density gradient centrifugation and frozen in liquid nitrogen for later RNA extraction. RNA was isolated from frozen PBMC using the Direct-zol RNA Miniprep kit (Zymo International) according to the manufacturer’s modified protocol for “RNA purification from the aqueous phase after Tri Reagent extraction.” Briefly, PBMC were mixed with 500 µL Trizol and further lysed using a bead mill. 100 µL of chloroform was added to the lysate, mixed well and centrifuged. The aqueous phase was removed, mixed with an equal volume of 100% ethanol and added to a Zymo-Spin IICR column. After centrifugation and a quick wash, on-column DNA digestion was performed, followed by additional washes. Total RNA was eluted with 35µL RNAse/DNAse free water and 1 µg was submitted to Canopy Biosciences (Hayward, CA) for quality testing (Qubit and Bioanlyzer) and NanoString nCounter analysis. DV200 values were above 40% for all samples and RIN values ranged from 2 to 9.

Gene expression analysis of 780 genes (Supplementary Table 1) was performed using the NanoString nCounter Canine Immuno-oncology (IO) Panel (Seattle, WA, USA). Data was analyzed using NanoString’s nSolver Analysis Software v4.0 with Advanced Analysis Module v2.0 plugin and included differential gene expression (DGE) analysis, gene set analysis (GSA), and cell type profiling. For cell type profiling analysis, we utilized the cell type scores to analyze the relative immune cell abundance levels across the time points in this study. The cell type score is calculated as the mean of the log2 expression levels for the probes linked to the specific cell type of interest. From this calculation, the cell type abundance in a single sample cannot be quantified, but the relative cell abundance level across time points or conditions can be analyzed. Here, we evaluated differences in cell type scores in the patient PBMCs across Days 0, 7, and 21. Patient samples were paired across timepoints for DGE analysis, and statistical significance was determined through the Benjamini-Hochberg method.

### Statistical design and power analysis

A sample size of ten was estimated to provide 80% power to detect an overall response rate (ORR) change from 11 to 50% using conditioning chemotherapy, a difference between our historical ORR (the null hypothesis) and the higher ORR of interest at the 0.05 level (1-sided). Using this study design, it is unlikely that modest improvements in ORR would be detected. Graph generation and statistical analysis were performed through GraphPad Prism software (v10.2.3). Kaplan-Meier curves and log-rank tests were utilized to examine survival outcomes. Statistical significance was defined as *p* < 0.05.

## Results

### Clinical characteristics

Ten dogs were enrolled in the study, including five dogs with OSA, four with melanoma, and one with multilobular tumor of bone (MLB). Of the four dogs with melanoma, prior to clinical trial enrollment, two had failed previous immunotherapy with the ONCEPT® Canine Melanoma Vaccine (Patient 205 and 210), two had surgery to remove the primary tumor (Patient 207 and 210), and one received palliative radiation (Patient 210). Following the clinical trial, one dog with melanoma received palliative radiation on Day 35 (Patient 208). In the five dogs with bone tumors, prior to enrollment, four underwent surgery to remove the primary tumor and received carboplatin treatment course; the other dog received palliative radiation (Patient 201). Histological or cytological confirmation of the primary tumor was available in all dogs. Patient characteristics are summarized in Table 1.

**Table 1:** Patient demographics and response

PatientID​	Weight(kg)​	Age(years)​	Sex​	Breed​	Disease​	Primary Tumor Location​	DoxorubicinDose ​	BestResponse​	Overall Survival (days)​	Progression-Free Survival (days)​
201​	37.1​	7​	MC​	Labrador Retriever​	OSA​	Left proximal tibia​	30 mg/m2​	NE​	26​	21​
202​	32.7​	5​	MC​	German Shepherd​	OSA​	Left distal radius​	30 mg/m2​	PD​	113​	35​
203​	28.6​	8​	FS​	Australian Cattle Dog​	OSA​	Fifth digit on right thoracic limb​	30 mg/m2​	SD​	127​	49​
204​	30.6​	8​	FS​	Pitbull​	MLB​	Maxilla​	30 mg/m2​	SD​	557​	77​
205​	8.9​	11​	FS​	Labrador mix​	MEL​	Left maxillary lip​	1 mg/kg​	PD​	>208​	35​
206​	46.5​	8​	FS​	Cane Corso​	OSA​	Left proximal humerus​	30 mg/m2​	NE​	42​	22​
207​	34.6​	10​	MC​	Giant Schnauzer​	MEL​	4th digit on left pelvic limb​	30 mg/m2​	PD​	127​	35​
208​	36.9​	9​	FS​	Golden Retriever​	MEL​	Right maxilla​	30 mg/m2​	CR​	>645​	645​
209​	26.85​	11​	FS​	Golden Retriever​	OSA​	Right proximal tibia​	30 mg/m2​	PD​	166​	35​
210​	7.9​	14​	FS​	Australian Shepherd​	MEL​	Left mandible​	1 mg/kg​	PD​	99​	35​

*MC* Male castrated, *FS *Female spayed, *MEL *Melanoma, *OSA *Osteosarcoma, *MLB *Multilobular tumor of bone, *NE *Not evaluable, *CR *Complete response, *PD* Progressive disease, *PR *Partial response, *SD *Stable disease

>still alive. Survival (days) was measured from Day 0 of clinical trial

### Adverse events

All AEs during the 21-day treatment course were captured and are provided in Supplementary Table 2. One patient experienced a serious AE following doxorubicin, developing a grade 4 neutropenia and grade 2 fever, which resolved within 2 days following hospitalization and supportive care. No other treatment-related serious AEs (grade 3 or above) were attributed to either the doxorubicin or inhaled rhIL-15 treatments. Overall, preconditioning doxorubicin did not alter the toxicity compared to the initial phase 1 trial [8].

### Hematologic changes

**Fig. 2:**
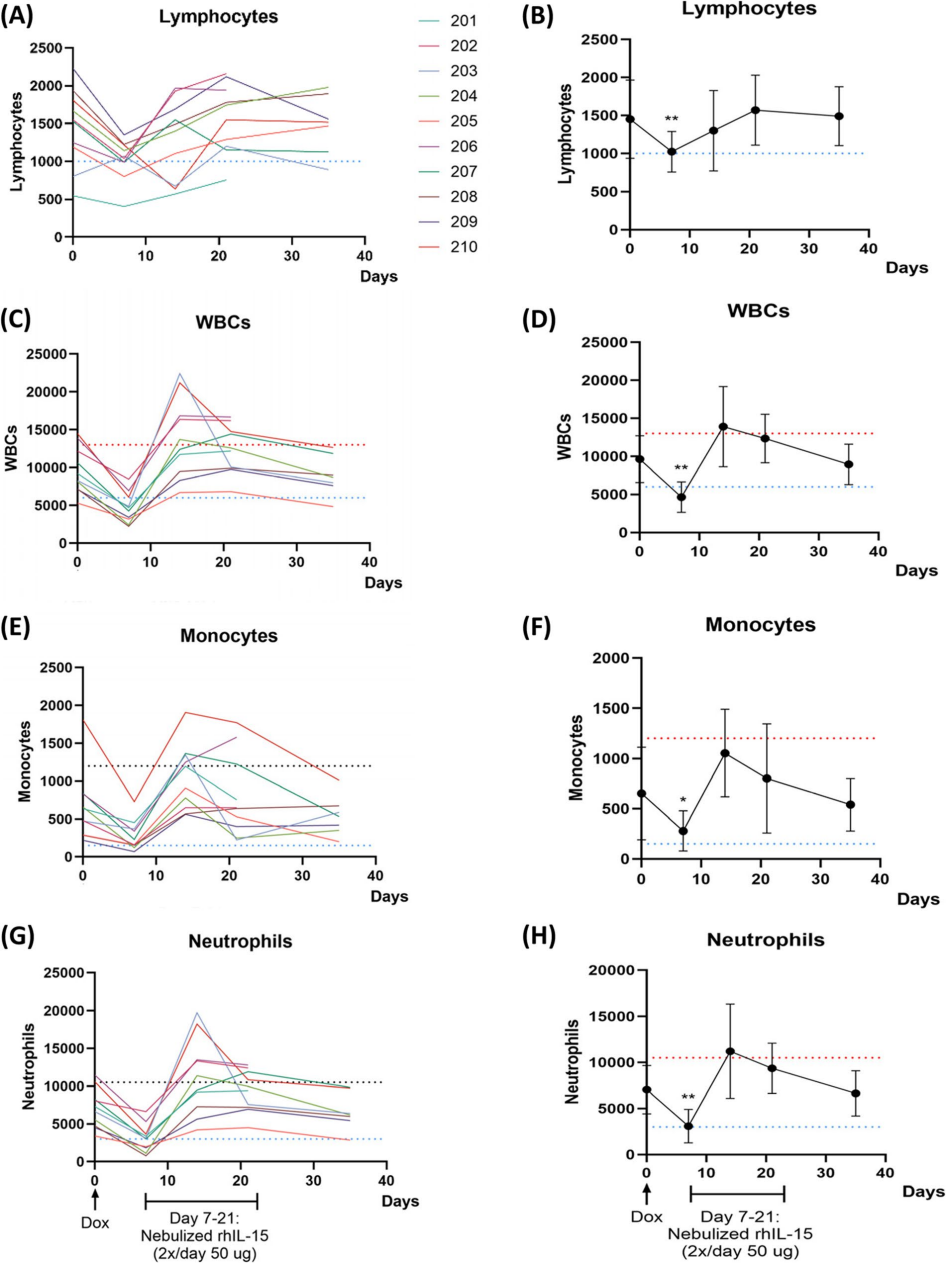
CBC values demonstrate significant reduction of WBC, neutrophils, lymphocytes, and monocytes seven days after doxorubicin treatment. Bloodwork was performed at baseline (Day 0), and then at Days 7, 14, 21 and 35. CBC values for (**A**) lymphocytes, (**C**) white blood cells (WBCs), (**E**) monocytes, and (**G**) neutrophils were tracked in all patients. The blue and red dashed lines denote the value that is classified as abnormally low or high, respectively, for each cell type. Doxorubicin *Dox* induced a significant decrease in (**B**) lymphocyte count (*p* < 0.01), (**D**) WBCs (*p* < 0.01), (**F**) monocytes (*p* < 0.05), and (**H**) neutrophils (*p* < 0.01) on Day 7. All CBC parameters increased after inhaled rhIL-15 treatment, then plateaued to baseline levels. A student’s unpaired, two-tailed t-test was utilized to determine statistical significance between Day 7 and Day 0

The objective of this study was to determine if lymphocyte reduction would improve response to inhaled rhIL-15. Hematologic parameters were evaluated to confirm doxorubicin resulted in lympho-reduction. The ALC significantly decreased 7 days after doxorubicin administration (Day 7) compared to baseline (Day 0), declining from a mean ALC of 1452 to 1023 cells/µL (*p* < 0.01) (Fig. 2A, B). In addition to ALC reduction, all patients exhibited lower leukocyte counts (*p* < 0.01), and monocyte counts (*p* < 0.05) on Day 7 (Fig. 2C-F). Lower neutrophil counts were observed in nine out of ten patients (*p* < 0.01), including one patient with grade 1 neutropenia and one patient described above with grade 4 neutropenia (Fig. 2G, H).

### Clinical responses and outcomes

**Fig. 3:**
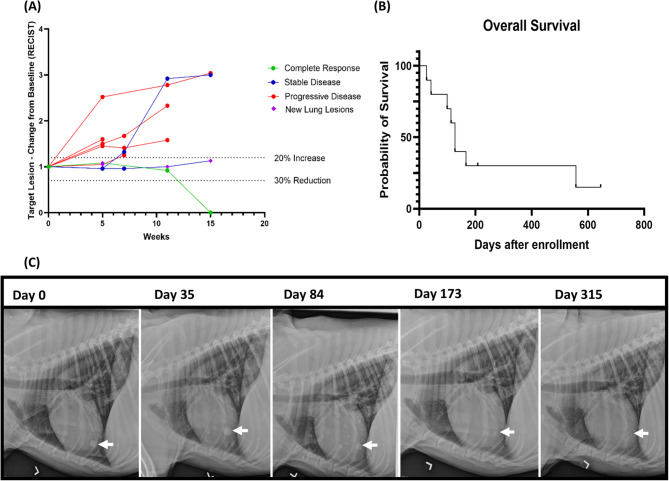
Preconditioning doxorubicin does not alter clinical response to inhaled rhIL-15. **A** Spider plot summarizing changes in the sum of each patient’s longest tumor diameters from baseline over time. Evaluable patients were classified by the best response as follows: complete response (green), stable disease (blue), or progressive disease (red) based on RECIST criteria. Stable disease was defined as the tumor diameter remaining within the thresholds of < 20% increase and < 30% decrease for a minimum of 28 days. A diamond shape demarcates the time new lung metastases were identified on radiographs. **B **Kaplan-Meier survival curve illustrating overall survival (OS) from inhaled rhIL-15 treatment and preconditioning doxorubicin in all patients. The median OS was 127 days and the median PFS was 35 days. **C** Radiographs demonstrating changes in pulmonary lesions before and after doxorubicin and inhaled rhIL-15 treatment in Patient 208. This patient initially demonstrated pseudo-progression on Day 35, followed by regression on Day 84 and a complete resolution of lung metastases by Day 173. This complete response has been maintained for > 2 years after the completion of treatment

Two dogs were withdrawn from the study before Day 35 due to the clinical progression of the disease, resulting in eight patients being evaluated for response outcomes. One dog achieved a complete response (CR), two had stable disease (SD), and five had progressive disease (PD) for an ORR of 10% and an overall CBR of 30%. Figure 3A summarizes the radiographically evaluable patient time points utilizing the RECIST criteria. Patient 208, a dog with melanoma, initially exhibited new radiographic lung lesions compatible with pulmonary metastatic disease at Day 35, and thus was classified as having unconfirmed PD. The dog then received palliative radiation (6 Gy once weekly for six weeks, starting on Day 35 and ending Day 77, for a total dose of 36 Gy delivered via IG-VMAT and 6MV photons) to the primary tumor. No other systemic therapy was given to this patient. By week 15, all lung lesions had resolved, and the dog was reclassified as having achieved a CR (Fig. 3C). This CR has been maintained for over 2 years, with no recurrence of the primary or metastatic lesions. Two dogs with SD maintained SD for 7 weeks (Patient 203) and 11 weeks (Patient 204), when new lung metastases were identified. The remaining five dogs exhibited PD, with four dogs having PD of target lesions by Day 35 and one having new lung lesions identified on Day 35. At the time of manuscript submission, eight dogs had died from OSA- or melanoma-related causes, while two dogs remained alive. Seven out of the eight dogs were euthanized due to progressive disease or decreased quality of life per the owner. It is unknown whether the other dog died from euthanasia or progression of disease. The median overall survival (OS) was 127 days (range = 26, > 645) for all dogs (Fig. 3B), 113 days (range 26–166) for dogs with OSA, 167.5 days (range 99, > 645 days, 2/4 alive) for dogs with melanoma, and 557 days for the dog with MLB. For all dogs in this current study, the median PFS was 35 days (range = 35, > 645) (Supplementary Fig. 1).

### NanoString transcriptomics

Using the NanoString nCounter Canine IO Panel, we next analyzed the differential gene expression (DGE) and cell type abundance measurements between patient PBMC collected at Day 0 (pre-doxorubicin), Day 7 (post-doxorubicin), and Day 21 (post-rhIL-15). Through analyzing the proportion of immune cell types – namely T cell subtypes, NK cells, and B cells – we sought to determine whether doxorubicin would induce indiscriminate lympho-reduction at Day 7 and whether inhaled rhIL-15 immunotherapy increased the proportion of cytotoxic cells at Day 21.

**Fig. 4:**
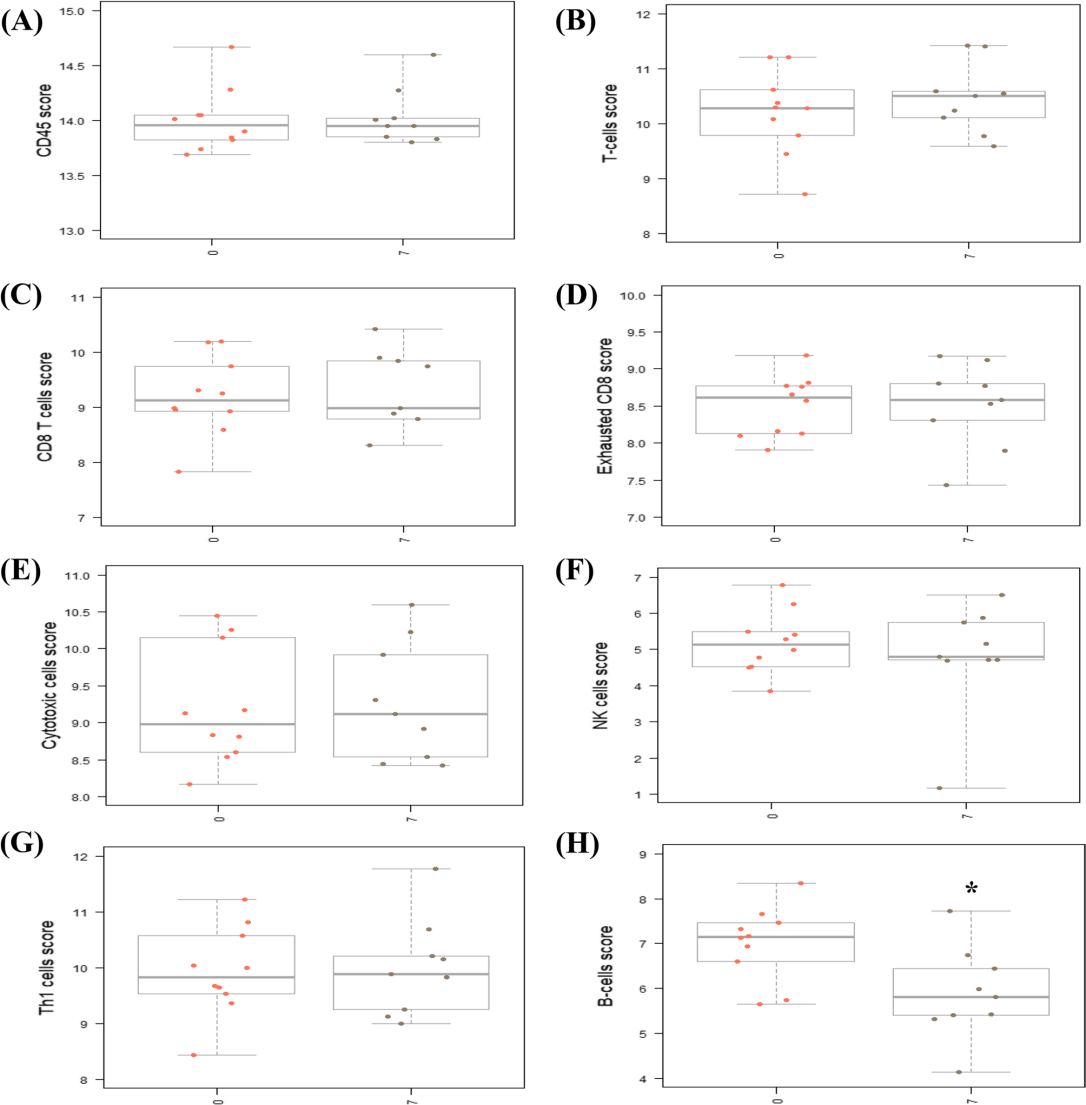
NanoString analysis of patient PBMC samples demonstrates a reduction in B-lymphocyte populations following doxorubicin. NanoString nCounter platform advanced analysis cell type profiling of patient PBMC samples on Day 7 and 0 using the Benjamini-Hochberg method to determine statistical significance. The cell score is calculated as the mean of the log2 expression levels for the probes linked to the specific cell type of interest. Presented is the mean and standard deviation of cell scores on Day 7 vs. Day 0 for the following cell types— (**A**) CD45, (**B**) T-cells, (**C**) CD8 T cells, (**D**) exhausted CD8 T cells, (**E**) cytotoxic cells, (**F**) natural killer (NK) cells, and (**G**) T helper-1 (Th1) cell. No significant difference in cell type proportions were observed from Day 0 to Day 7. **H** The B cell score was significantly decreased (*p* < 0.05) from Day 0 to Day 7. A student’s unpaired, two-tailed t-test was utilized to determine statistical significance

Using pairwise analysis to evaluate DGE, there were 244 upregulated genes and 338 downregulated genes in PBMC populations following doxorubicin treatment (Day 7), relative to baseline (Day 0) (Supplementary Table 3, Supplementary Fig. 2 A). Next, we analyzed cell type scores to assess relative immune cell population abundance levels in all dogs from Day 0 to Day 7. No difference was observed in the proportion of hematopoietic cells, based on scores determined for CD45 + cells (Fig. 4A). Similarly, no significant changes in cell type scores were observed for total T cells, CD8 + T cells, exhausted CD8 + T cells, cytotoxic cells, Th1 cells, or NK cells from Day 0 to Day 7 (Fig. 4B-G, Supplementary Fig. 3 A). As we observed a significant decrease in absolute lymphocyte count from the CBC results, this finding indicates that although doxorubicin reduced the ALC, the T-lymphocyte proportion was not significantly altered. These results are consistent with previous findings that standard doses of doxorubicin induce indiscriminate lymphodepletion in dogs [18]. Interestingly, however, there was a significant decrease in the B cell score of PBMC on Day 7 relative to Day 0 (Fig. 4H, *p* < 0.05). Among the top differentially expressed genes (DEGs) from Day 7 vs. Day 0, multiple genes associated with B cell function— namely, IGHG, CD79B, BLK, MS4A1, and CD19— were significantly downregulated (Table 2, Supplementary Fig. 4 A, *p* < 0.01). These findings indicate that doxorubicin may differentially deplete the B cell population.

**Table 2:** Top 20 Down- and upregulated differentially expressed genes (DEGs) in PBMCs in dogs before and after treatment with doxorubicin (Day 7 vs. Day 0). NanoString nCounter platform differential gene expression analysis of paired patient PBMC samples on Day 7 and 0 using the Benjamini-Hochberg method to determine statistical significance

** Day 7 vs. 0 Top 20 DEGs**				
Gene Symbol	Log2 fold change	Std error (log2)	Adjusted p-value	NanoString gene sets
Downregulated				
CAMP	−2.83	0.104	2.02e-06	Angiogenesis
IGHG	−2.27	0.141	6.42e-05	B-Cell Functions, Lymphoid Compartment
POU2AF1	−1.73	0.157	0.000804	
TNFRSF17	−1.7	0.264	0.00762	Cell Functions, Cytokine And Chemokine Signaling, NF-kB Signaling, TNF Superfamily
BLK	−1.51	0.155	0.00116	B-Cell Functions, Lymphoid Compartment
LTF	−1.48	0.235	0.00773	
CD19	−1.38	0.193	0.00488	B-Cell Functions, Complement System, Lymphoid Compartment, PI3K-Akt, Regulation
CD79B	−1.36	0.132	0.00101	B-Cell Functions, Lymphoid Compartment
MS4A1	−1.1	0.134	0.00261	B-Cell Functions, Lymphoid Compartment
ICOSLG	−1.01	0.112	0.00152	Cell Functions, Costimulatory Signaling, Immune Cell Adhesion And Migration, Lymphoid Compartment, Regulation
FCER2	−0.792	0.115	0.00573	Cytokine And Chemokine Signaling
BLNK	−0.789	0.124	0.00762	Cytokine And Chemokine Signaling, NF-kB Signaling
PTGDR2	−0.755	0.115	0.00712	Cell Functions
CX3CR1	−0.745	0.078	0.00116	Angiogenesis, Chemokines, Cytokine And Chemokine Signaling, Microglial Functions
CFD	−0.742	0.116	0.00762	Complement System
MS4A2	−0.676	0.111	0.00904	Chemokines
CD79A	−0.624	0.0799	0.00302	Lymphoid Compartment
ITGB2	−0.514	0.0854	0.00907	Adhesion, Angiogenesis, Cytokine And Chemokine Signaling, Immune Cell Adhesion And Migration, Matrix Remodeling And Metastasis, Regulation
Upregulated				
MSR1	0.965	0.123	0.00302	Cell Functions
TREM2	1.04	0.145	0.00488	Myeloid Compartment

**Fig. 5:**
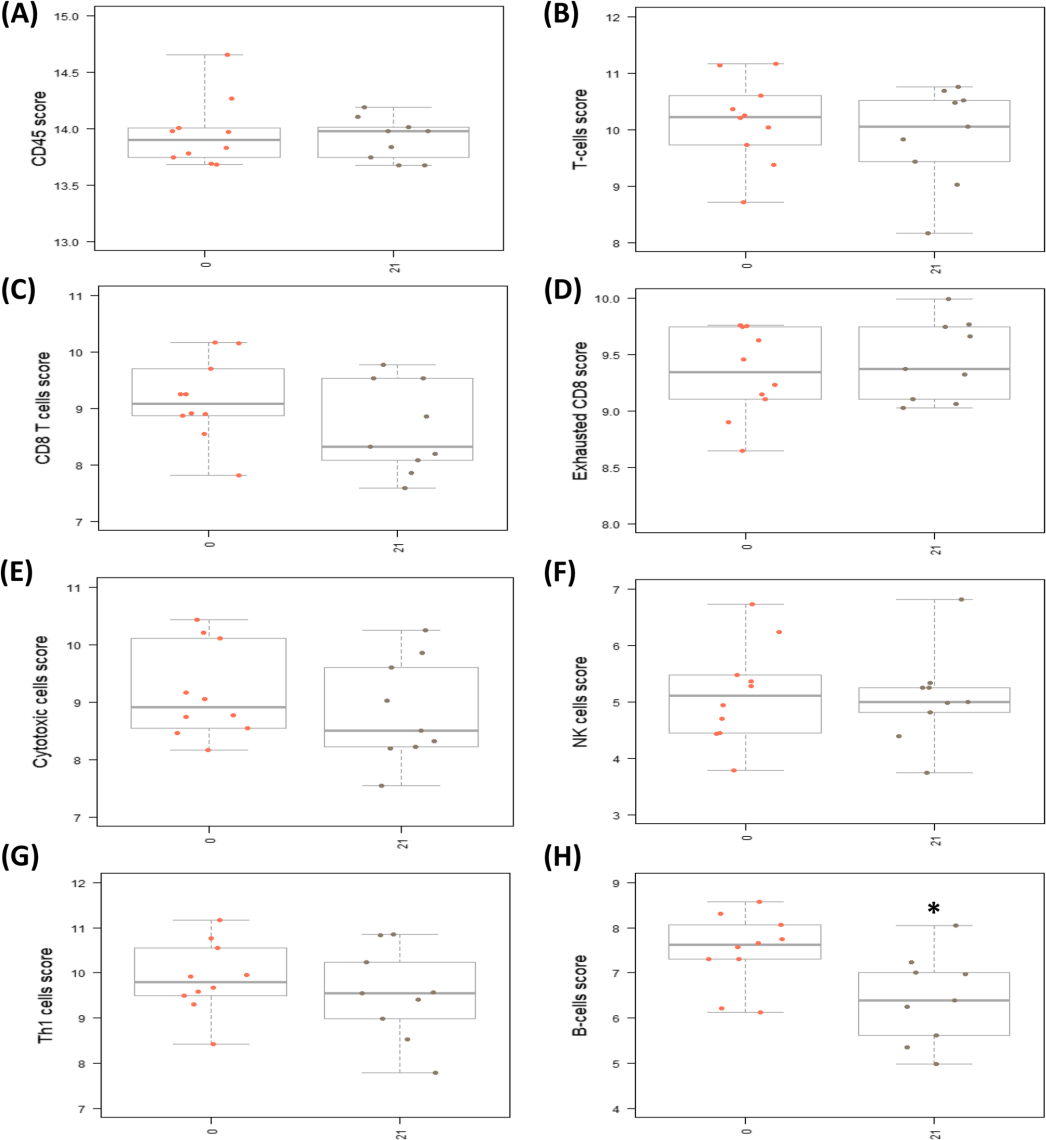
NanoString analysis of patient PBMC samples demonstrates no significant alteration in T and NK cell type score following 2 weeks of inhaled rhIL-15 treatment. NanoString nCounter platform advanced analysis cell type profiling of patient PBMC samples on Day 21 and 0 using the Benjamini-Hochberg method to determine statistical significance. The cell score is calculated as the mean of the log2 expression levels for the probes linked to the specific cell type of interest. Presented is the mean and standard deviation of cell scores on Day 21 vs. Day 0 for the following cell types— (**A**) CD45, (B) T-cells, (**C**) CD8 T cells, (**D**) exhausted CD8 T cells, (**E**) cytotoxic cells, (**F**) natural killer (NK) cells, and (**G**) T helper-1 (Th1) cell. No significant difference in cell type proportions were observed from Day 0 to Day 21. (**H**) The B cell score was significantly decreased (*p* < 0.05) from Day 0 to Day 21. A student’s unpaired, two-tailed t-test was utilized to determine statistical significance

Next, we evaluated DGE and cell type abundance of PBMC collected on Day 21 compared to Day 0. Utilizing pairwise analysis, there were 257 upregulated genes, and 318 downregulated genes in PBMC populations following doxorubicin treatment and two weeks of inhaled rhIL-15 immunotherapy (Day 21), relative to baseline (Day 0) (Supplementary Table 4, Supplementary Fig. 2B). Similar to the cell type score analysis on Day 7, no difference in cell type scores was observed for CD45 + cells, T cells, CD8 + T cells, exhausted CD8 + T cells, cytotoxic cells, Th1 cells, or NK cells from Day 0 to Day 21 (Fig. 5A-G, Supplementary Fig. 3B). No difference in cell type scores was observed for these immune cell types between Day 21 and Day 7 (data not shown). Interestingly, the B cell score and associated gene expression profile remained significantly lower on Day 21, relative to Day 0 (Fig. 5H; Table 3, Supplementary Fig. 4B). This result indicates that the two weeks of inhaled rhIL-15 immunotherapy did not reverse the doxorubicin-induced B-cell population depletion observed on Day 7.

Given that no differences were observed in the proportion of T cells or NK cells on Day 21 when evaluating all patients together, we sought to determine whether we could observe differences in the evaluated cell type scores in the subset of patients who exhibited clinical benefit in this trial. When analyzing the two dogs with stable disease and the one dog with a CR, we found that there was no significant alteration in the proportion of CD45 + cells, T cells, CD8 + T cells, exhausted CD8 + T cells, cytotoxic cells, Th1 cells, or NK cells on Day 7 vs. Day 0 or Day 21 vs. 0 (Supplementary Fig. 5A-G, 6 A-G). The B cell score was significantly decreased in responder patients on Day 7 and Day 21, as was consistent with the cell type score analysis conducted on all patients (Supplementary Fig. 5H, 6 H). To further elucidate whether transcriptomic changes in the PBMC populations would correlate with clinical benefit, we analyzed the cell type score of T-cells, CD8 + T cells, cytotoxic cells, and exhausted CD8 + T cells on Days 0, 7, and 21 in patients segregated based on response (Supplementary Fig. 7A-D). Interestingly, the single dog with a CR trended towards a higher cell score for T cells, CD8 + T cells, and cytotoxic cells relative to dogs with PD or SD. The patient with a CR also had a low exhausted CD8 + T cell score on Day 0 but was at similar levels to dogs with PD or SD on Days 7 and 21. This observation could support the notion that response to inhaled rhIL-15 therapy may be associated with the proportion of effector immune cell types at baseline. However, no conclusions can be made based on a single observation.

## Discussion

Based on the previous phase 1 trial evaluating inhaled rhIL-15 in dogs with metastatic tumors of bone or melanoma, we expected to observe an improved ORR to inhaled rhIL-15 with the addition of doxorubicin. However, preconditioning doxorubicin resulted in a similar ORR (10%) and CBR (30%) compared to inhaled rhIL-15 treatment alone (11% and 39%, respectively) [8]. Five dogs exhibited PD, two had SD that lasted 7- and 11-weeks from Day 0, and one demonstrated complete resolution of diffuse lung metastases for over 2 years. Overall, these findings suggest that preconditioning doxorubicin does not improve the clinical efficacy of inhaled rhIL-15 immunotherapy in dogs with melanoma or sarcoma.

As observed in our previous trials, no allergic reaction or anaphylaxis was seen, and no pulmonary-related AEs were attributed to inhaled rhIL-15 [8, 18]. One patient demonstrated grade 4 neutropenia with a grade 2 fever on Days 8–9 following doxorubicin administration. After hospitalization, symptoms resolved within 48 h and were attributed to doxorubicin-induced chemotoxicity [19]. Of note, the dog that experienced grade 4 febrile neutropenia was the dog that went on to experience a CR [8]. No other serious AEs were attributed to either doxorubicin or inhaled rhIL-15 treatment, thus indicating that preconditioning with doxorubicin did not demonstrably increase the toxicity of inhaled rhIL-15 in this small population of dogs.

Next, we evaluated the changes in the immune cell type population following doxorubicin. Hematological parameters have been demonstrated to correlate with response to therapy in canine cancer patients [20, 21]. Our previous phase 1 trial in dogs showed a correlation between low baseline ALC (a mean ALC of 1040/µL vs. 1485/µL) and clinical response to inhaled rhIL-15 [8]. Doxorubicin has previously been shown to reduce T cells and ALC in dogs [22]. Based on CBC results, we demonstrate that doxorubicin significantly reduced lymphocyte, monocyte, and neutrophil populations at Day 7. Comparable to the ALC levels in the phase 1 trial, the mean ALC for dogs at Day 0 was 1452/µL, and the median ALC at Day 7 was 1023/µL. In other words, preconditioning doxorubicin reduced the ALC to comparable levels to what was observed in the responding dogs from the phase 1 trial, per the objective of this study. However, this was not associated with increased treatment efficacy (ORR or CBR). In the current study, neither a low baseline ALC nor a low ALC induced by doxorubicin was associated with clinical benefit. Therefore, this study failed to confirm the previously reported correlation between a low baseline ALC and response to rhIL-15. While we hoped that doxorubicin would result in a “reset” of the lymphocyte population that could be more responsive to inhaled IL-15, one concern is that lympho-reduction secondary to doxorubicin could lead to reduced efficacy of rhIL-15 as there could be a reduction in cytotoxic and NK cells for the IL-15 to stimulate. Nevertheless, we demonstrated no difference in ORR or CBR with the addition of preconditioning doxorubicin in this trial.

In addition to hematological changes, we evaluated the differential gene expression and immune cell type population changes following doxorubicin and inhaled rhIL-15 therapy. NanoString transcriptomic analysis of patient PBMCs revealed no differences in the proportion of CD8 + T cell, Th1 cell, NK cell, or cytotoxic cell population levels following doxorubicin treatment on Day 7. Specifically, CBC results confirmed the overall lymphocyte count decreased following doxorubicin, but transcriptomic analysis demonstrated no selective decrease of any specific T-lymphocyte subset. A limitation of the NanoString platform is we could not directly evaluate differences in the population frequencies of T-regulatory cells. A previous study, however, has demonstrated doxorubicin induces T cell lymphodepletion without selectivity for T-regulatory cells [22]. Interestingly, we found that the B cell type score and B cell function genes were notably decreased following doxorubicin treatment. These results indicate that while doxorubicin overall reduced WBCs, including lymphocytes, the lymphocyte-reduced fraction may preferentially deplete a higher proportion of B-cells than T-cells. One explanation could be that B cells proliferate at a more rapid rate than T-cell subtypes and were thereby more impacted by the doxorubicin treatment [23, 24]. As the B-cell type score remained low by Day 21, this reflects that the B cells did not repopulate to their pre-treatment levels as quickly as the other evaluated cell populations post-chemotherapy. Previously, it has been demonstrated in human patients that B cells dramatically deplete following chemotherapy and remain at lower levels for months afterwards [25]. Flow cytometry to characterize the circulating lymphocyte populations before and after doxorubicin would be valuable in confirming RNA expression results in future studies.

Lastly, we anticipated that inhaled rhIL-15 immunotherapy would increase the proportion of cytotoxic cells, including CD8 + T and NK cells. However, NanoString transcriptomic analysis of all patient PBMCs revealed that there were no differences in the proportion of CD8 + T cell, Th1 cell, NK cell, or cytotoxic cell population levels following inhaled rhIL-15 immunotherapy on either Day 7 or Day 21, compared to Day 0. We previously demonstrated low systemic exposure in dogs treated with inhaled rhIL-15. Therefore, it is not surprising that inhaled rhIL-15 may result in minimal systemic changes in PBMC populations [8, 26, 27]. However, it is interesting to note that when analyzing responder and non-responder populations across Days 0, 7, and 21, the CR patient had consistently higher T cell, CD8 + T cell, and cytotoxic cell scores than those with PD or SD. Additionally, the CR patient had a lower exhausted CD8 + T cell score than the other patients on Day 0. This finding could support the notion that T cell exhaustion may be a contributing factor in resistance to inhaled rhIL-15 therapy. These results are interesting as they indicate differential responses following inhaled rhIL-15, which correlates with prior ex-vivo cytotoxicity data in the phase 1 trial [8]. However, data from a single dog in this study should be interpreted cautiously.

Several limitations of this study should be noted. First, rhIL-15 was used, rather than recombinant canine IL-15. While we did not observe any allergic or anaphylactic reactions to human IL-15, it is likely to result in the production of neutralizing antibodies, which may further limit the efficacy of this treatment. Consistent with our previous studies using inhaled human IL-15 in dogs [8, 18], we chose to treat dogs for only two weeks, as we felt it was unlikely for significant neutralizing antibodies to be generated in this short time frame. Another key limitation of this trial was its small sample size, especially since two of the ten enrolled patients did not survive to Day 35, which excluded them from formal response rate evaluation. We also deviated from the RECIST criteria to include patients with target lung metastatic lesions measuring less than 2 cm. This criteria was consistent with our previous published phase 1 study [8] and was based on our experience that dogs with > 2 cm metastatic lung lesions often have advanced clinical disease and do not meet eligibility and/or performance score criteria. Nevertheless, this represents a weakness, as radiographic measurements can be imprecise and may limit the reproducibility of the data [8].

Despite these limitations, three patients exhibited clinical benefit, supporting further research into optimizing inhaled rhIL-15 immunotherapy. However, other factors may have influenced these responses. Tumor type could have played a role in response to therapy. One of the dogs with SD had a primary tumor identified as MLB, a slow-progressing bone cancer. This characteristic may explain this patient’s slow progression of pulmonary nodules [28]. In both OSA and melanoma, survival outcomes can vary based on the primary tumor location [29, 30]. However, given the small sample size in this study, our study was not designed or powered to detect any differences in prognosis based on the original primary tumor location within each tumor type. Generally, once pulmonary metastatic disease is present, OSA and melanoma carry similarly poor prognoses of less than 80 days [31, 32]. Additionally, target lesions were lung metastases, and we did not track potential responses in primary tumors, which represents a limitation and could be an interesting future direction.

The dog that achieved a CR was initially classified as having unconfirmed PD due to new lesions identified on Day 35 thoracic radiographs. At that time, the owners elected to withdraw the dog from the study to pursue palliative radiation for the primary tumor located on the lower right mandible. We hypothesized that the new radiographic lesions observed after the completion of inhaled rhIL-15 therapy at Day 35 represented pseudoprogression (PP), emphasizing the importance of keeping PP in the differential diagnosis following immunotherapy treatment [33, 34]. Another option is that the radiation to the primary tumor could have induced an abscopal effect independent of the inhaled rhIL-15. Abscopal responses, however, are rarely reported in patients receiving radiation alone [35]. Radiation in combination with immunotherapy, interestingly, has demonstrated clinical responses in dogs with OSA [36] and dogs with malignant melanoma [37]. However, it is unclear whether this played a role in this patient’s clinical response. Further investigation into the potential benefit of combining inhaled rhIL-15 immunotherapy with radiation therapy would need to be conducted.

## Conclusion

Preconditioning with doxorubicin did not improve the clinical response to inhaled rhIL-15 immunotherapy in dogs with metastatic tumors of bone or melanoma. Three out of ten dogs experienced clinical benefit. While doxorubicin induced indiscriminate lymphodepletion, it did not alter the response rate or toxicity profile of inhaled rhIL-15. These findings confirm that durable responses occur in a subset of dogs with advanced metastatic disease and support continued investigation into optimizing inhaled rhIL-15 immunotherapy for canine patients with metastatic sarcoma or melanoma.

## Supplementary Information

Supplementary Material 1.

Supplementary Material 2.

Supplementary Material 3.

Supplementary Material 4.

## Data Availability

NanoString results are provided as supplemental information. All other data is available at request.

## Abbreviations

OSA: Osteosarcoma
MLB: multilobular tumor of bone
rhIL15: recombinant human IL-15
VCOG-CTCAE v2: Veterinary Cooperative Oncology Group-Common Terminology Criteria for Adverse Events
AE: Adverse events
NK: Natural killer
ALC: Absolute lymphocyte count
NSAIDs: Non-steroidal anti-inflammatory drugs
CBC: Complete blood count
RECIST: Response evaluation criteria for solid tumors in dogs
SD: Stable disease
PR: Partial response
CR: Complete response
PP: Pseudo-progression
PFS: Progression free survival
OS: Overall survival
PBMCs: Peripheral blood mononuclear cells
CBR: Clinical benefit rate
ORR: Overall response rate
DGE: Differential gene expression
DEG: Differentially expressed genes
