# Supplementary material for: Evaluating the impact of doxorubicin preconditioning on the efficacy of inhaled recombinant human IL-15 immunotherapy in dogs with pulmonary metastasis
Source: Vet Oncol. 2025 Oct 1;2(1):23. doi: 10.1186/s44356-025-00040-5 (PMC12484353; doi:10.1186/s44356-025-00040-5)
Supplement: Supplementary file 2 — Supplementary Material 2. [file 44356_2025_40_MOESM2_ESM.pptx]

## Slide 1
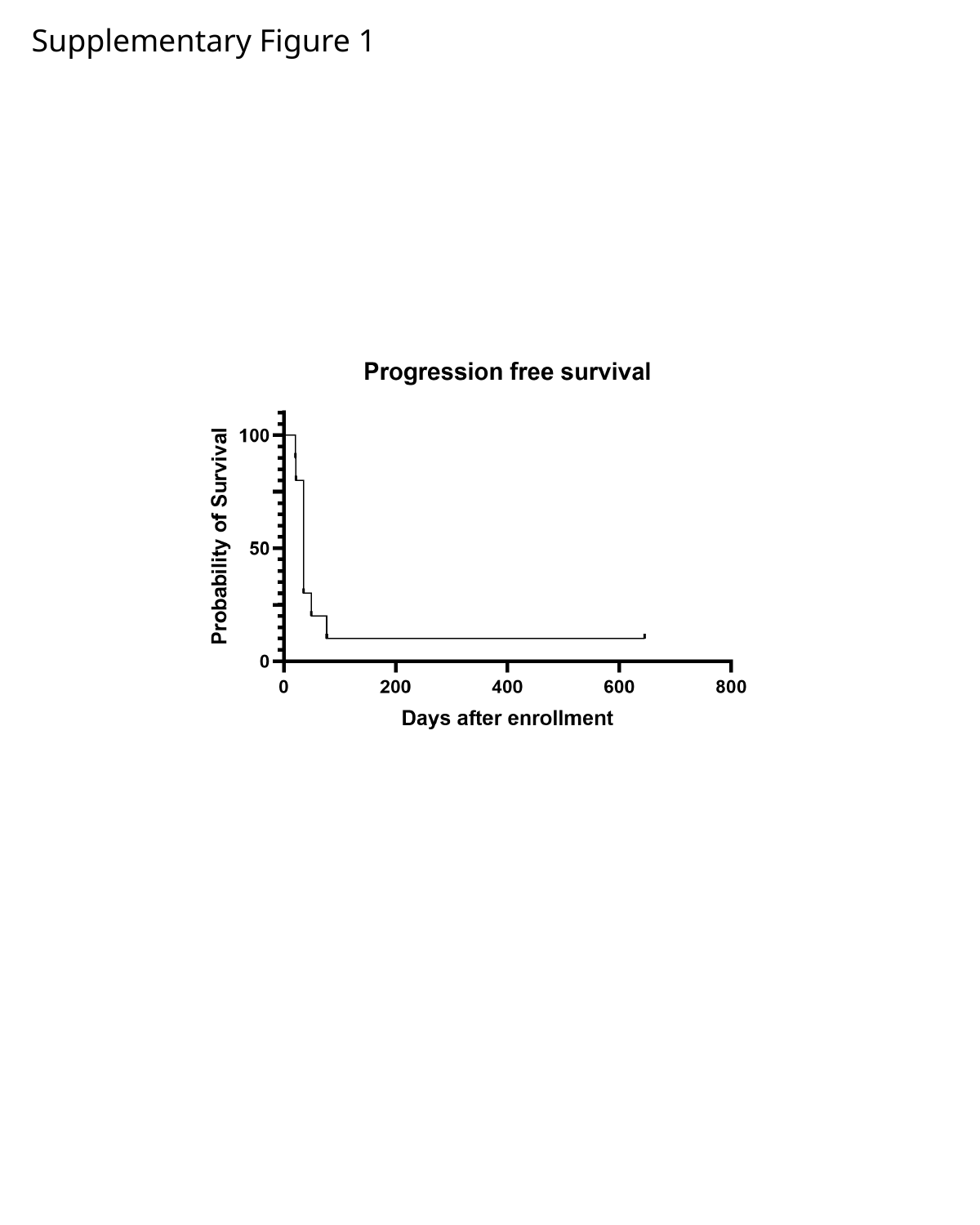

Supplementary Figure 1

## Slide 2
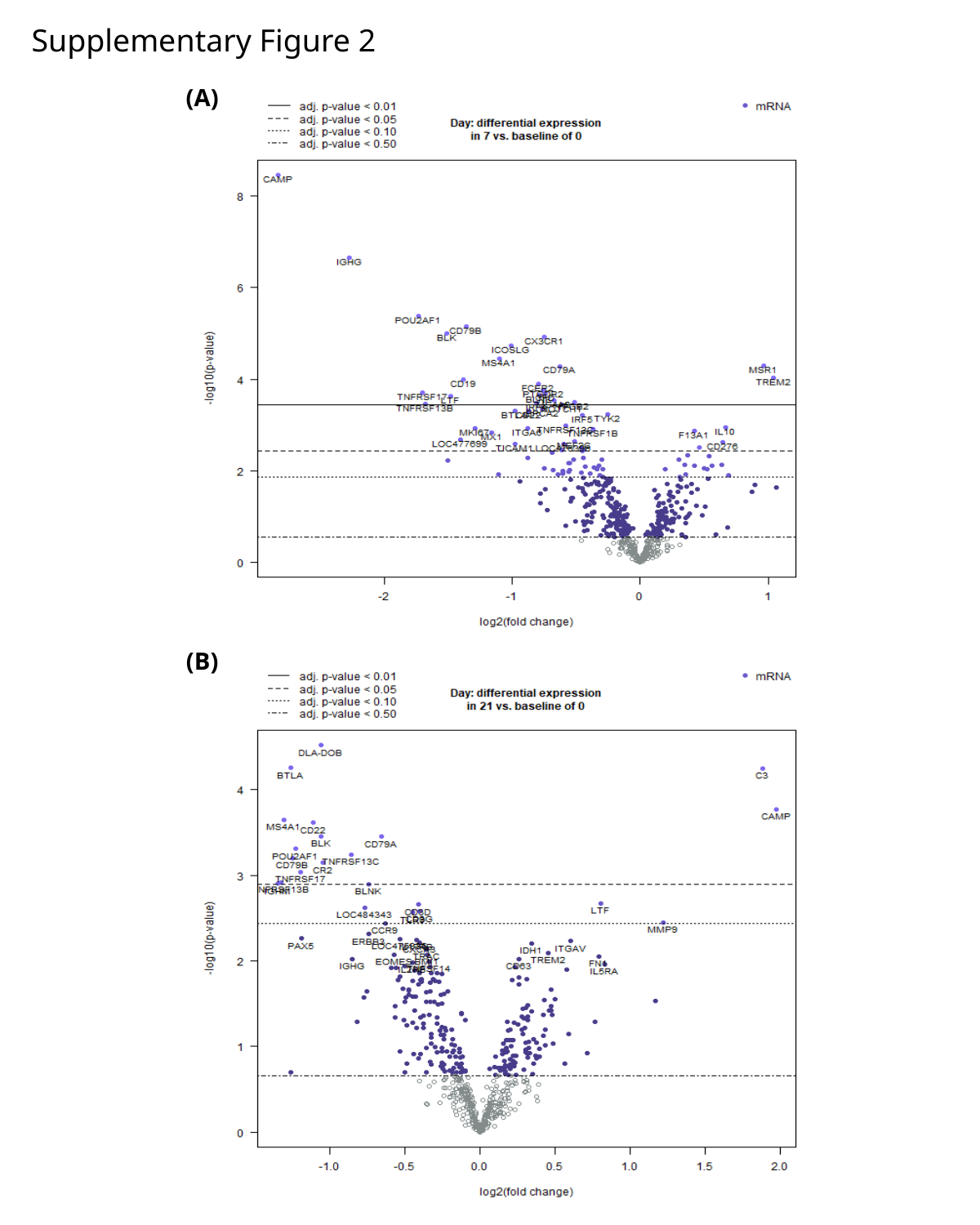

Supplementary Figure 2
(A)
(B)

## Slide 3
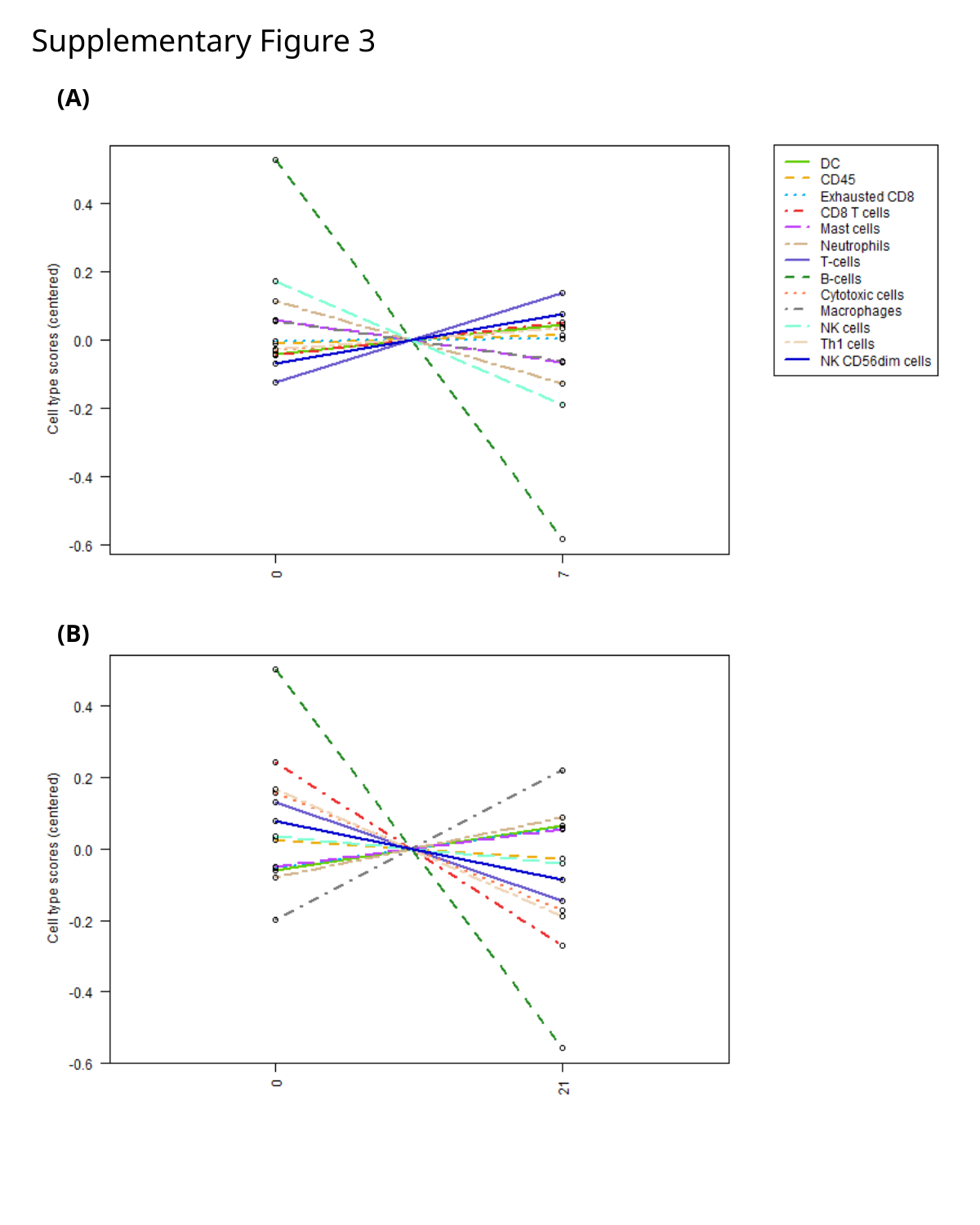

Supplementary Figure 3
(A)
(B)

## Slide 4
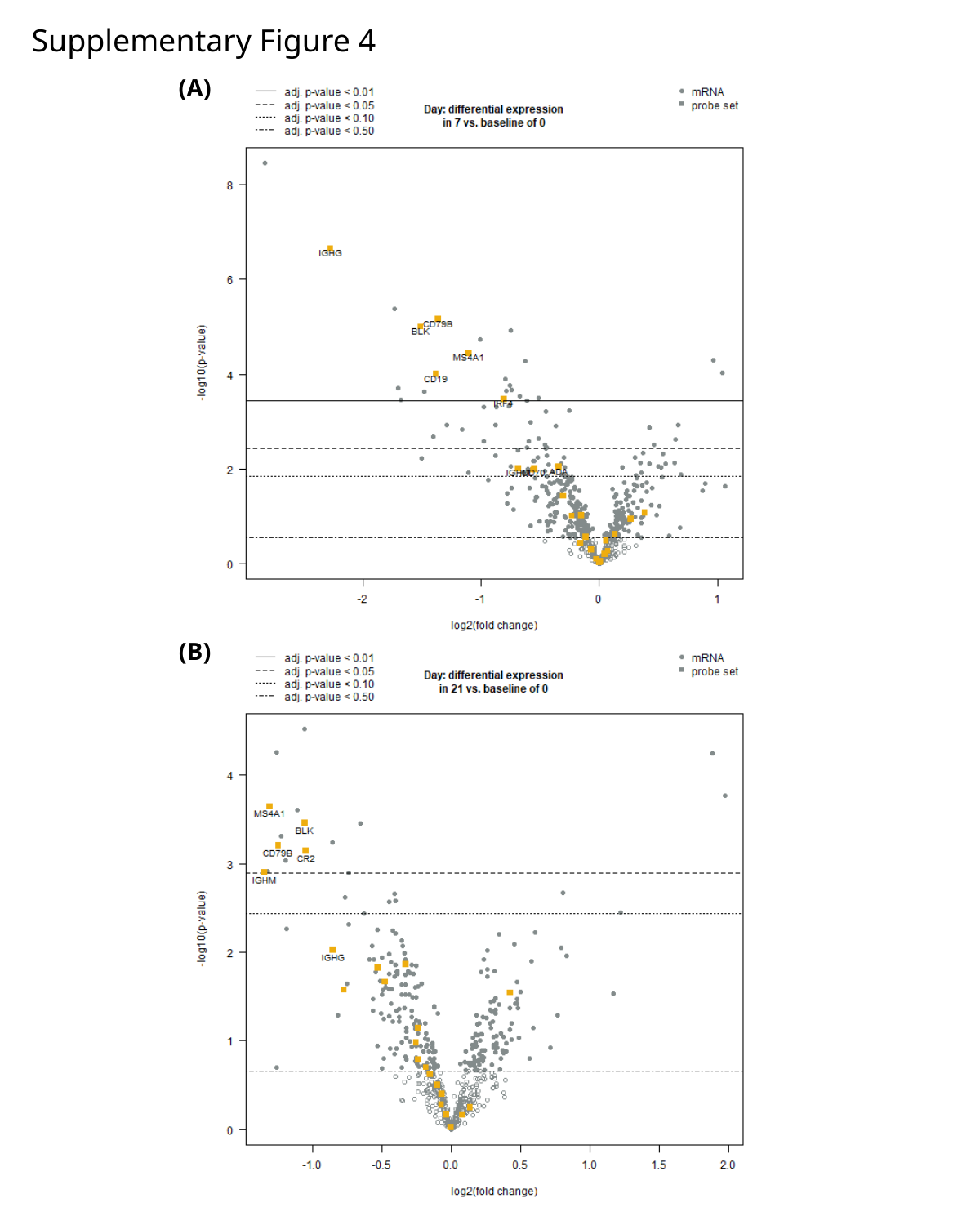

Supplementary Figure 4
(A)
(B)

## Slide 5
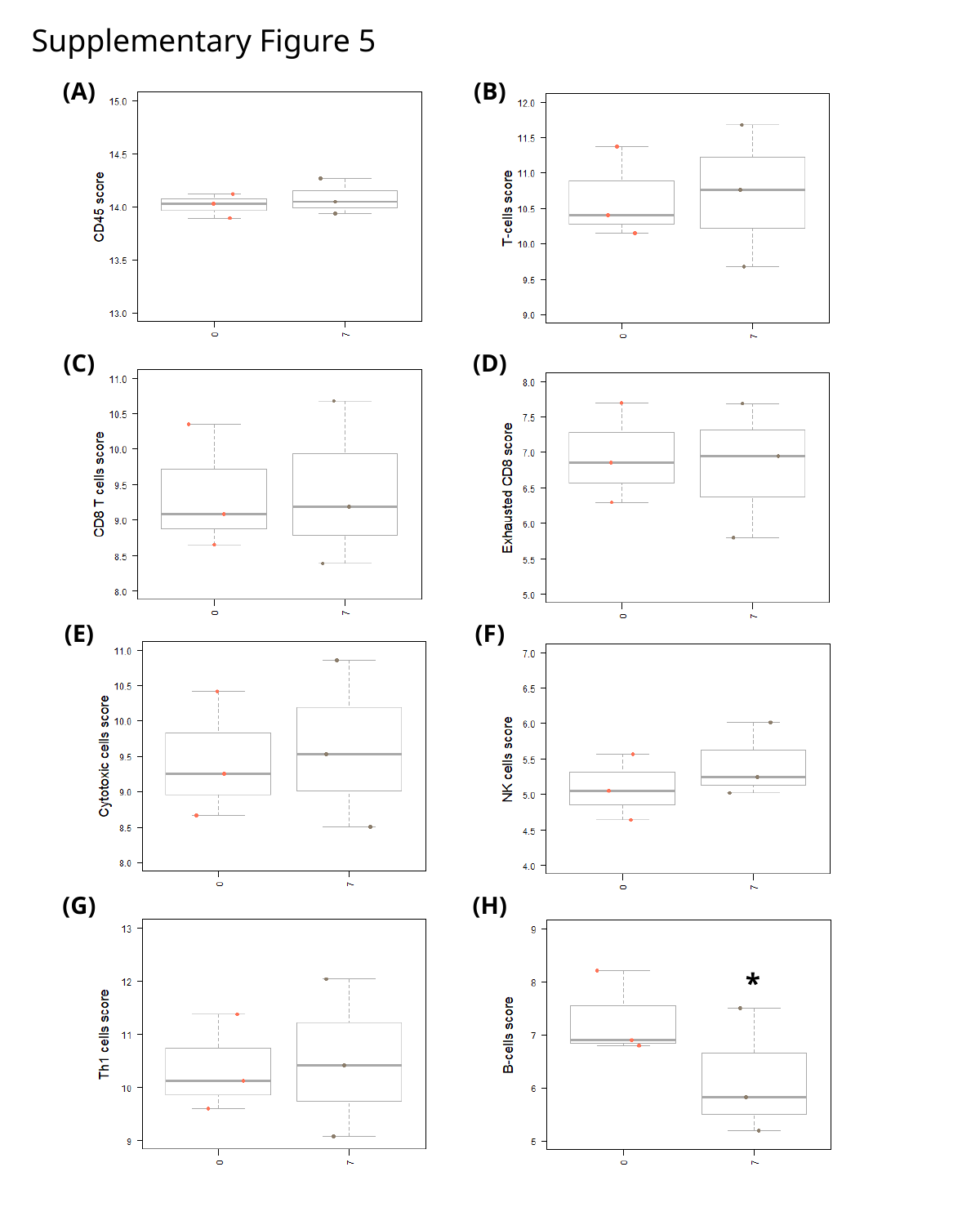

Supplementary Figure 5
(A)
(B)
(C)
(D)
(E)
(F)
(G)
(H)
*

## Slide 6
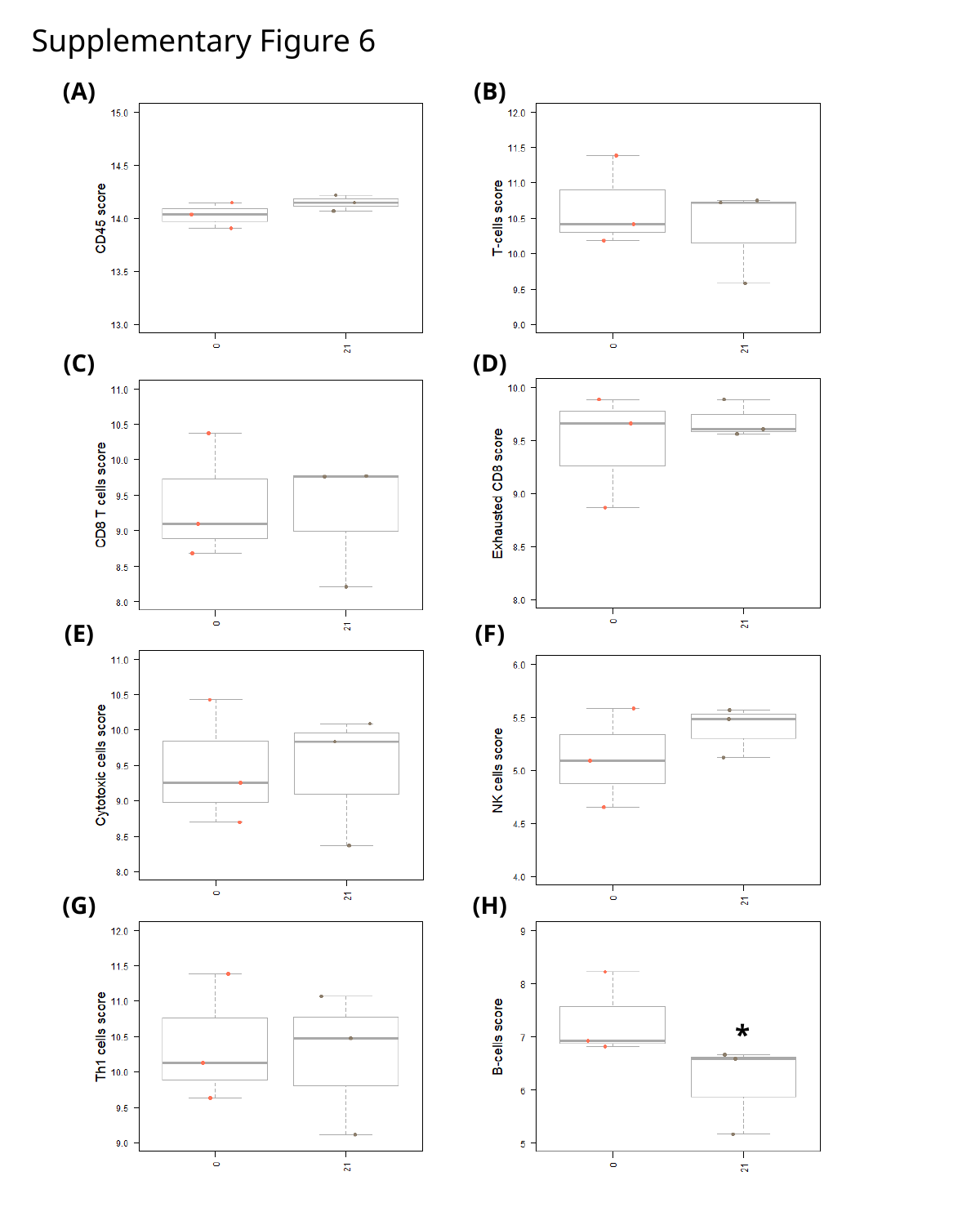

Supplementary Figure 6
(A)
(B)
(C)
(D)
(E)
(F)
(G)
(H)
*

## Slide 7
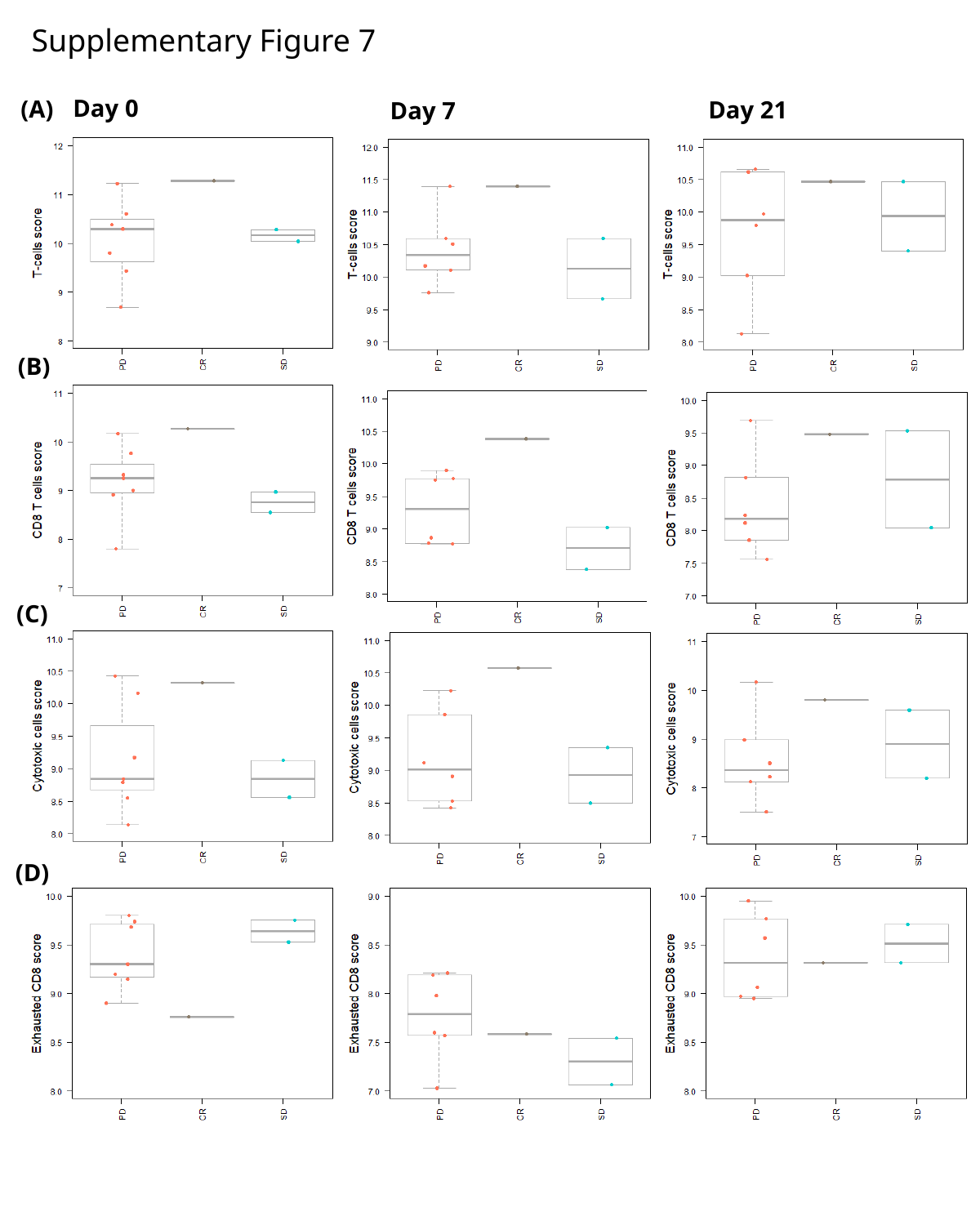

Supplementary Figure 7
Day 0
Day 21
Day 7
(A)
(B)
(C)
(D)

## Slide 8
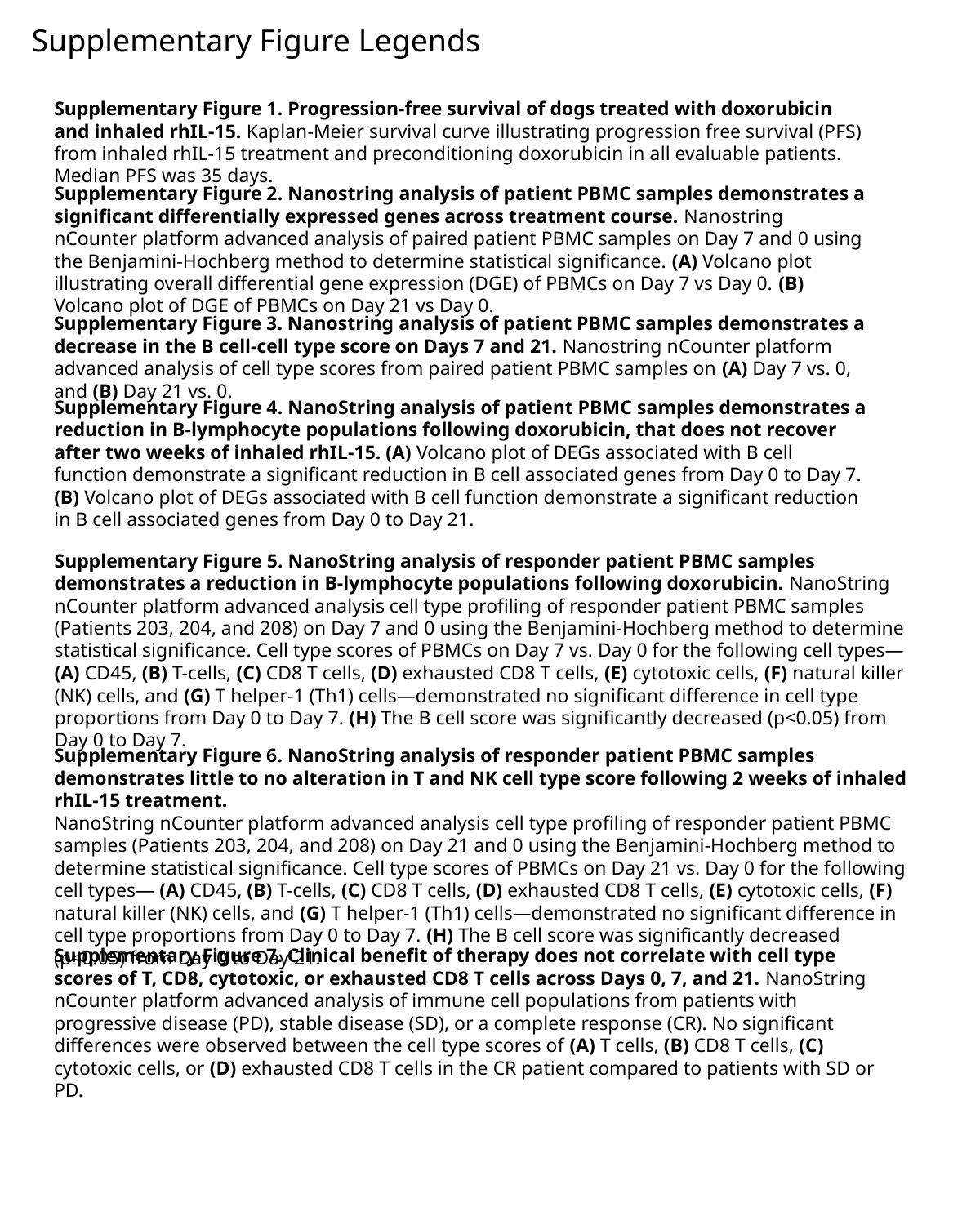

Supplementary Figure Legends
Supplementary Figure 1. Progression-free survival of dogs treated with doxorubicin and inhaled rhIL-15. Kaplan-Meier survival curve illustrating progression free survival (PFS) from inhaled rhIL-15 treatment and preconditioning doxorubicin in all evaluable patients. Median PFS was 35 days.
Supplementary Figure 2. Nanostring analysis of patient PBMC samples demonstrates a significant differentially expressed genes across treatment course. Nanostring nCounter platform advanced analysis of paired patient PBMC samples on Day 7 and 0 using the Benjamini-Hochberg method to determine statistical significance. (A) Volcano plot illustrating overall differential gene expression (DGE) of PBMCs on Day 7 vs Day 0. (B) Volcano plot of DGE of PBMCs on Day 21 vs Day 0.
Supplementary Figure 3. Nanostring analysis of patient PBMC samples demonstrates a decrease in the B cell-cell type score on Days 7 and 21. Nanostring nCounter platform advanced analysis of cell type scores from paired patient PBMC samples on (A) Day 7 vs. 0, and (B) Day 21 vs. 0.
Supplementary Figure 4. NanoString analysis of patient PBMC samples demonstrates a reduction in B-lymphocyte populations following doxorubicin, that does not recover after two weeks of inhaled rhIL-15. (A) Volcano plot of DEGs associated with B cell function demonstrate a significant reduction in B cell associated genes from Day 0 to Day 7. (B) Volcano plot of DEGs associated with B cell function demonstrate a significant reduction in B cell associated genes from Day 0 to Day 21.
Supplementary Figure 5. NanoString analysis of responder patient PBMC samples demonstrates a reduction in B-lymphocyte populations following doxorubicin. NanoString nCounter platform advanced analysis cell type profiling of responder patient PBMC samples (Patients 203, 204, and 208) on Day 7 and 0 using the Benjamini-Hochberg method to determine statistical significance. Cell type scores of PBMCs on Day 7 vs. Day 0 for the following cell types— (A) CD45, (B) T-cells, (C) CD8 T cells, (D) exhausted CD8 T cells, (E) cytotoxic cells, (F) natural killer (NK) cells, and (G) T helper-1 (Th1) cells—demonstrated no significant difference in cell type proportions from Day 0 to Day 7. (H) The B cell score was significantly decreased (p<0.05) from Day 0 to Day 7.
Supplementary Figure 6. NanoString analysis of responder patient PBMC samples demonstrates little to no alteration in T and NK cell type score following 2 weeks of inhaled rhIL-15 treatment.
NanoString nCounter platform advanced analysis cell type profiling of responder patient PBMC samples (Patients 203, 204, and 208) on Day 21 and 0 using the Benjamini-Hochberg method to determine statistical significance. Cell type scores of PBMCs on Day 21 vs. Day 0 for the following cell types— (A) CD45, (B) T-cells, (C) CD8 T cells, (D) exhausted CD8 T cells, (E) cytotoxic cells, (F) natural killer (NK) cells, and (G) T helper-1 (Th1) cells—demonstrated no significant difference in cell type proportions from Day 0 to Day 7. (H) The B cell score was significantly decreased (p<0.05) from Day 0 to Day 21.
Supplementary Figure 7. Clinical benefit of therapy does not correlate with cell type scores of T, CD8, cytotoxic, or exhausted CD8 T cells across Days 0, 7, and 21. NanoString nCounter platform advanced analysis of immune cell populations from patients with progressive disease (PD), stable disease (SD), or a complete response (CR). No significant differences were observed between the cell type scores of (A) T cells, (B) CD8 T cells, (C) cytotoxic cells, or (D) exhausted CD8 T cells in the CR patient compared to patients with SD or PD.
