# Supplementary material for: Evaluating the impact of doxorubicin preconditioning on the efficacy of inhaled recombinant human IL-15 immunotherapy in dogs with pulmonary metastasis
Source: Vet Oncol. 2025 Oct 1;2(1):23. doi: 10.1186/s44356-025-00040-5 (PMC12484353; doi:10.1186/s44356-025-00040-5)
Supplement: Supplementary file 4 — Supplementary Material 4. [file 44356_2025_40_MOESM4_ESM.pdf]

INTRAVENOUS DOXORUBICIN AND INHALED IL-15 IMMUNOTHERAPY FOR TREATMENT OF LUNG METASTASES

VCCT STUDY No. 22-009

IACUC No. 22674

Regulatory Sponsor: NIH/NCI

Study Product: IL-15

Principal Investigator: Robert Rebhun
[redacted]

Co-Investigators: Michael Kent
[redacted]

Version History
Original Version Date: 24AUG2022
Version Number: 1.0
Amendment Date: 27OCT2022

Table of Contents

CONFIDENTIAL
This material is the property of the University of California. Do not disclose or use except as authorized in writing by the study sponsor

STUDY SUMMARY 1
1 INTRODUCTION 2
1.1 BACKGROUND 2
1.2 INVESTIGATIONAL AGENT 2
1.3 RATIONALE AND RISK/BENEFITS 2
2 STUDY OBJECTIVES 2
3 STUDY DESIGN 3
3.1 GENERAL DESIGN 3
3.2 PRIMARY STUDY ENDPOINTS 3
3.3 SECONDARY STUDY ENDPOINTS 3
3.4 PRIMARY SAFETY ENDPOINTS 3
4 STUDY SITE(S), STAFF, AND FACILITIES 3
4.1 STUDY SITE(S) 3
4.2 STUDY STAFF 3
4.2.1 Contact Information 3
4.3 STUDY FACILITIES 4
4.3.1 Clinical Research Area 4
4.3.2 Document Storage 4
4.3.3 Laboratory Services 4
4.3.4 Research Sample Storage 4
5 SUBJECT SELECTION AND WITHDRAWAL 4
5.1 INCLUSION CRITERIA 4
5.2 EXCLUSION CRITERIA 5
5.3 SUBJECT RECRUITMENT 5
5.4 SUBJECT SCREENING 5
5.4.1 Screening Procedures 5
5.4.2 Determination of Eligibility 6
5.4.3 Informed Consent 6
5.5 SUBJECT IDENTIFICATION 6
5.6 SUBJECT RETENTION 6
5.7 EARLY WITHDRAWAL OF SUBJECTS 6
5.7.1 When and How to Withdraw Subjects 6
5.7.2 Data Collection and Follow-up for Withdrawn Subjects 6
5.7.3 Lost to Follow-up 6
6 STUDY INVESTIGATIONAL PRODUCT 7
6.1 DESCRIPTION 7
6.2 TREATMENT REGIMEN 7
6.3 ASSIGNING SUBJECTS TO TREATMENT GROUPS 7
6.4 PREPARATION AND ADMINISTRATION OF INVESTIGATIONAL PRODUCT 7
6.5 PACKAGING 8
6.6 BLINDING OF INVESTIGATIONAL PRODUCT 8

CONFIDENTIAL
This material is the property of the University of California Do not disclose or use except as authorized in writing by the study sponsor

6.7 RECEIVING, STORAGE, DISPENSING AND RETURN 8
6.7.1 Receipt of Investigational Product 8
6.7.2 Storage 8
6.7.3 Dispensing of Investigational Product 8
6.7.4 Return or Destruction of Investigational Product 8

7 STUDY PROCEDURES 9

7.1 SCHEDULE OF EVENTS 9
7.2 VISIT REQUIREMENTS 10
7.3 TRIAL COMPLIANCE 12
7.3.1 Prior and Concomitant Therapy 12
7.3.2 Lifestyle/Diet Considerations 13
7.3.3 Subject Compliance Monitoring 13
7.3.4 Protocol Deviations 13
7.4 CLINICAL PROCEDURES / ASSESSMENTS 13
7.5 LABORATORY PROCEDURES / ASSESSMENTS 13

8 STATISTICAL PLAN 13

8.1 SAMPLE SIZE DETERMINATION 13
8.2 STATISTICAL METHODS 13
8.3 SUBJECT POPULATION(S) FOR ANALYSIS 14

9 SAFETY AND ADVERSE EVENTS 14

9.1 DEFINITIONS 14
9.2 RECORDING OF ADVERSE EVENTS 15
9.3 REPORTING OF SERIOUS ADVERSE EVENTS AND UNANTICIPATED PROBLEMS. 15
9.3.1 Investigator reporting: notifying the study sponsor 15
9.4 STOPPING RULES 16
9.5 MEDICAL MONITORING 16

10 DATA HANDLING AND RECORD KEEPING 16

10.1 CONFIDENTIALITY 16
10.2 SOURCE DOCUMENTS 16
10.3 PARTICIPANT BINDER 17
10.4 CASE REPORT FORMS 17
10.5 ELECTRONIC DATA CAPTURE 17
10.6 RECORDS RETENTION ..... **ERROR! BOOKMARK NOT DEFINED.**

11 STUDY MONITORING, AUDITING, AND INSPECTING 18

11.1 STUDY MONITORING 18
11.2 AUDITING AND INSPECTING 18

12 ETHICAL CONSIDERATIONS 18

13 STUDY COMPLETION AND CLOSEOUT PROCEDURES 18

14 STUDY FINANCES 19

14.1 FUNDING SOURCE 19
14.2 CONFLICTS OF INTEREST 19

This material is the property of the University of California Do not disclose or use except as authorized in writing by the study sponsor

14.3 SUBJECT STIPENDS OR PAYMENTS 19

15 PUBLICATION PLAN 19

16 VETERINARY CENTER FOR CLINICAL TRIALS STANDARD OPERATING PROCEDURES 20

16.1 INTRAVENOUS DOXORUBICIN AND INHALED IL-15 IMMUNOTHERAPY FOR TREATMENT OF LUNG METASTASES 20

17 REFERENCES 20

18 ATTACHMENTS 20

List of Abbreviations

AE – Adverse event
CRF – Case report form
CTCAE – Common terminology and criteria for adverse events
CTRB - Clinical Trials Review Board
EDC - Electronic Data Capture
FDA – Food and Drug Administration
GCP - Good Clinical Practice
HIPAA – Health information portability and accountability act
IACUC – Institutional Animal Care and Use Committee
PHI – Protected health information
PI – Principal investigator
SAE – Serious adverse event
SOP - Standard operating procedure
VCCT - Veterinary Center for Clinical Trials
VCOG – Veterinary cooperative oncology group
VMTH - Veterinary medical teaching hospital

CONFIDENTIAL
This material is the property of the University of California Do not disclose or use except as authorized in writing by the study sponsor

Study Summary

Title	Intravenous Doxorubicin and Inhaled IL-15 Immunotherapy for Treatment of Lung Metastases
Short Title	Inhaled IL-15 plus Doxorubicin
VCCT Study No	VCCT-22-009
Methodology	Single-armed study
Study Duration	1 year
Recruitment Duration	1 year
Study Center(s)	UC Davis VMTH
Objectives	To determine whether preconditioning lymphodepletion with doxorubicin will improve response rates to inhaled IL-15 in dogs with lung metastases. To determine toxicity and tolerability.
Number of Subjects	10
Diagnosis and Main Inclusion Criteria	Dogs with pulmonary metastasis greater than 1cm.
Study Product, Dose, Route, Regimen	Recombinant Human Interleukin-15 (IL-15), 50ug, inhalation, twice daily for 2 weeks. Doxorubicin (30mg/m2 or 1mg/kg depending on clinician prescription), IV on Day 0.
Duration of participation	98 Days
Reference therapy	Doxorubicin
Outcome measures	Laboratory test values, physical examination, owner questionnaire.

CONFIDENTIAL
This material is the property of the University of California. Do not disclose or use except as authorized in writing by the study sponsor

1 Introduction

This document is a protocol for a dog research study. While companion animal trials are not covered under the US and international standards of Good Clinical Practice (FDA Title 21 part 312 and International Conference on Harmonization guidelines), many of the principles apply. This study is to be conducted according to these standards where applicable and according to government regulations and Institutional research policies and procedures.

1.1 Background

Previous trials have found that inhaled IL-15 is well tolerated and resulted in durable responses in dogs with metastatic melanoma or osteosarcoma. Analyses of correlative data found that dogs with low lymphocyte counts were more likely to achieve clinical benefit from this therapy. The goal for this study is to determine whether lymphodepletion with doxorubicin prior to initiating inhaled IL-15 might improve response rates in canine patients with metastatic tumors to the lung. The primary endpoints of this study are to determine the safety and preliminary evidence of efficacy for this combination treatment. Toxicity, response, and progression will be evaluated based on modified versions of the VCOG consensus documents for adverse events and RECIST criteria for solid tumors.

1.2 Investigational Agent

Recombinant Human Interleukin-15 (IL-15) with standard doxorubicin chemotherapy

1.3 Rationale and Risk/Benefits

Dogs with lung metastases secondary to melanoma or osteosarcoma have a dismal prognosis and response rates to standard chemotherapy are less than 20% for most tumor types. Therefore, these patients are ideal candidates for novel therapies. Previous studies have demonstrated durable responses to inhaled IL-15 in dogs with metastatic osteosarcoma or melanoma without any recognized toxicity. If successful, this trial will provide important evidence in a large animal, outbred model of spontaneous metastasis that preconditioning chemotherapy with doxorubicin treatment followed by immunotherapy with inhaled IL-15 is a safe and potentially effective treatment for dogs with lung metastasis.

2 Study Objectives

2.1 Primary Objective

To determine whether preconditioning lymphodepletion with doxorubicin will improve response rates to inhaled IL-15 in dogs with lung metastases.

2.2 Secondary Objective

Toxicity and tolerability

3 Study Design

3.1 General Design

The trial is an open label unmasked non-randomized single-armed trial where all enrolled dogs will receive treatment. The trial will evaluate whether preconditioning lymphodepletion with doxorubicin will improve response rates to inhaled IL-15 in dogs with lung metastases.

The trial will consist of the following phases:
Screening, enrollment, lymphodepletion (doxorubicin administration), treatment with investigational agent (IL-15), assessment for response, long-term follow-up/monitoring for disease progression.

The expected duration of subject participation is 98 days, but may be longer if decided by the PI and pet owner.

3.2 Primary Study Endpoints

Objective response rates based on radiographic response

3.3 Secondary Study Endpoints

Response duration, toxicity

3.4 Primary Safety Endpoints

Based on VCOG and known toxicity of doxorubicin. Toxicity will be assessed using client completed forms along with physical exam, temperature, CBC, Chemistry panel, urinalyses.

4 Study Site(s), Staff, and Facilities

4.1 Study Site(s)

Single site; Veterinary Medical Teaching Hospital, School of Veterinary Medicine, University of California-Davis

4.2 Study Staff

4.2.1 Contact Information

Role	Name	Telephone	E-mail
PI	[redacted]	[redacted]	[redacted]
Co-I	[redacted]	[redacted]	[redacted]
Clinician	[redacted]	[redacted]	[redacted]
Lead Coordinator	[redacted]	[redacted]	[redacted]
Back-up Coordinator	[redacted]	[redacted]	[redacted]
Statistician			

CONFIDENTIAL
This material is the property of the University of California Do not disclose or use except as authorized in writing by the study sponsor

4.3 Study Facilities

4.3.1 Clinical Research Area

Veterinary Medical Teaching Hospital, School of Veterinary Medicine, University of California-Davis; CCAH

4.3.2 Document Storage

Paper CRF records in a study binder will be kept in Oncology. Following patient completion, the paper source records will be scanned and saved in a shared Box folder. Patient and client identifying information will be redacted. Additionally, UC Davis will maintain its own abbreviated REDCap database. The UCD REDCap will be used to electronically collect QOL surveys from pet owners. Adverse Events will also be entered into this database for ease of analysis. Basic patient and owner information (ID numbers, e-mails, etc.) will be included to facilitate submission of survey.

4.3.3 Laboratory Services

Routine laboratory tests will be submitted through the VMTH with study number for billing purposes.

4.3.4 Research Sample Storage

[N/A.]

5 Subject Selection and Withdrawal

5.1 Inclusion Criteria

<u>Canine:</u>
In order to be eligible for trial participation, the subject must:

1. Present with one or more pulmonary nodules measuring at least 1cm on radiographs consistent with metastatic disease from cytologically or histologically confirmed osteosarcoma or melanoma
2. Adequate local control of primary tumor (i.e. surgery or radiation)
3. Radiographs performed within 7 days of enrollment (Day 0)
4. Dogs > 1 year
5. Body Weight > 15 kg
6. VCOG-CTCAE v2 constitutional clinical signs < grade 2
7. Overseeing clinician deems dog able to undergo sedation for radiographs
8. CBC and Chemistry performed within 7 days of enrollment (Day 0) showing adequate organ function:
    a. HCT > 25%, Neutrophil Count > 2,000/ul, Platelet Count >75,000/ul,
    b. Creatinine < ULN; bilirubin < ULN; ALT< ULN; AST < ULN
9. Urinalysis performed within 7 days of enrollment (Day 0)
10. Owner consent for dog's inclusion into the trial

CONFIDENTIAL
This material is the property of the University of California Do not disclose or use except as authorized in writing by the study sponsor

Caretaker:

In order to be eligible for participation, the pet's caregiver must be:

1. ≥ 18 years of age on the day of signing the informed consent
2. Able to understand the requirements of the study as determined by the investigators or research assistants
3. Able to give informed consent for on behalf of the dog for inclusion in the study
4. Able to read, understand and fill out the questionnaires
5. Able to administer trial treatment to their dog as demonstrated at the first visit

5.2 Exclusion Criteria

1. Owner unable or unwilling to administer inhaled IL-15 twice daily and/or dog unable to tolerate twice daily nebulization
2. Prior IL-15 therapy
3. Chemotherapy within 2 weeks of Day 0 (prior doxorubicin is acceptable)
4. Immunotherapy or radiation therapy within 2 weeks of Day 0
5. Surgery within 2 weeks of Day 0
6. Known or suspected MDR mutation
7. Cardiac disease precluding doxorubicin as determined by the clinician
8. Concurrent therapy**
   8.1.**Exceptions: NSAIDS for pain control acceptable if patient has received for > 2 weeks, pamidronate or zoledronate also acceptable

5.3 Subject Recruitment

Subjects will be recruited internally through the UC Davis VMTH services and from targeted advertisements via referring hospitals, StudyPages, social media, and online listings. Dogs participating in this study will be privately owned.

5.4 Subject Screening

5.4.1 Screening Procedures

Patient medical records will be evaluated to determine eligibility. If the dog appears to meet preliminary inclusion criteria, a formal appointment/consultation (trials service) will occur. At which time, the owners will be given detailed information on the trial, including nebulization as well as standard therapy options. If interested, the owner will review the Informed Consent Form with trials staff and sign the document.

5.4.1.1 Subject Re-screening

If the PI and overseeing clinician determine that a patient may be eligible for re-screening at a later date (i.e. pulmonary nodules <1cm, patient or client require additional time to acclimate to the nebulizer, etc.), this will be determined on a case-by-case basis where the inclusion/exclusion criteria still apply as described and additional required diagnostics would be at the owner's expense.

CONFIDENTIAL
This material is the property of the University of California Do not disclose or use except as authorized in writing by the study sponsor

5.4.2 Determination of Eligibility

Determination of eligibility will be by the clinical trials clinician unless there are questions or concerns and then consultation with Dr. Rebhun will result in determination.

5.4.3 Informed Consent

Informed consent will be obtained by the study coordinator or trials clinician at or prior to the Week 0 visit after the study clinician has confirmed preliminary eligibility. The informed consent form is attached as an appendix. A copy of the signed consent form will be provided to the owner, one copy will be scanned into the patient medical record in VMACS on the visit for Day 0, and the original signed copy will be kept in the patient study binder.

5.5 Subject Identification

Dogs will be identified by their given name and owner's surname, as recorded in their medical records. In addition, dogs will be identified by both their assigned by their unique medical record number in VMACs, and by a study number that will consist of UCD-followed by a 4-digit sequential number starting with the number 201. e.g. UCD-IL15Doxo-201, UCD-IL15Doxo-202, UCD-IL15Doxo-203, etc. The study number is assigned on Day 0.

5.6 Subject Retention

Standard visit reminders, phone calls between visits, e-mail check-ins, calls from the coordinators.

5.7 Early Withdrawal of Subjects

5.7.1 When and How to Withdraw Subjects

Subjects may be withdrawn at the request of the caregiver, due to adverse events, and/or at the investigator's discretion. If any patient becomes increasingly sick, and the quality of life is of concern to the study staff, discussions will occur to determine how to proceed.

The investigator may remove the subject from the trial if their caregivers fail to bring them to scheduled study visits and/or provide data and the caregivers cannot be reached by the study site for 2 weeks. Alternatively, if caregiver cannot consistently give the patient the twice daily inhaled dose of IL-15, they may be removed from the study.

5.7.2 Data Collection and Follow-up for Withdrawn Subjects

Request permission for follow up phone calls or emails relating to outcome data. UCD can provide prepaid shipping label for return of nebulizer and supplies.

5.7.3 Lost to Follow-up

Subjects are considered lost to follow-up if their caregivers fail to bring them to scheduled study visits and/or provide data and the caregivers cannot be reached by the study site. Attempts will be made once a month for 3 months by phone and email prior to being deemed LTFU.

CONFIDENTIAL
This material is the property of the University of California Do not disclose or use except as authorized in writing by the study sponsor

6 Study Investigational Product

6.1 Description

Recombinant Human Interleukin-15 (IL-15)-E. Coli product is described by the NCI Drug Dictionary as:

A recombinant agent that is chemically identical or similar to the endogenous cytokine interleukin-15 (IL-15) with immunomodulating activity. IL-15, secreted by mononuclear phagocytes (and some other cell types) following viral infection, regulates T and natural killer cell activation and proliferation. This cytokine induces activation of transcription activators STAT3, STAT5, and STAT6 via JAK kinase signal transduction pathways in mast cells, T cells, and dendritic epidermal T cells. IL-15 and interleukin-2 (IL-2) are structurally similar and share many biological activities; both may bind to common hematopoietin receptor subunits, negatively regulating each other's activity. CD8+ memory T cell number has been shown to be regulated by a balance between IL-15 and IL-2.

Standard commercially available doxorubicin administered and prepared as per typical UCD-VMTH protocol.

6.2 Treatment Regimen

All enrolled dogs are in the treatment group and will receive doxorubicin (30mg/m2 or 1mg/kg depending on clinician prescription) IV on Day 0 and then 50ug inhaled IL-15 by nebulization twice daily x 14 days starting Day 7. No dose escalation to occur.

6.3 Assigning Subjects to Treatment Groups

N/A, single-arm study, no masking or randomization

6.4 Preparation and Administration of Investigational Product

A clinical trials coordinator will be responsible for the preparation and packaging of the IL-15

- This includes thawing and mixing the IL-15 and then drawing up into enough 6mL syringes for 14 days' worth at twice daily dosing
- The IL-15 will need to be packaged on ice for transport with care to avoid freeze/thaw.

A medical oncology staff member will be responsible for the preparation and administration of the doxorubicin.

6.4.1 Special Handling Instructions

Personal Protective Equipment (PPE) Required for IL-15 Handling (trials personnel & client):

- Disposable chemotherapy gown, disposable N95 mask, eye protection, and gloves. (PPE will be sent home with the client)
- Should only be handled and administered in a well-ventilated area
- Once prepared, IL-15 must be stored at 4°C for up to 14 days

Personal Protective Equipment (PPE) Required for doxorubicin Handling (trials & VMTH personnel):

  - Standard as per VMTH/medical oncology policies

  - Disposable chemotherapy gown, eye protection, and gloves.
- Should only be drawn up in the designated chemotherapy hood in medical oncology

6.5 Packaging
See Appendix

6.6 Blinding of Investigational Product
N/A

6.7 Receiving, Storage, Dispensing and Return

6.7.1 Receipt of Investigational Product
N/A

6.7.2 Storage
IL-15 must be stored at 4°C for up to 14 days.

6.7.3 Dispensing of Investigational Product
Clinical trials will be responsible for thawing and mixing the IL-15 and pulling up into 6mL syringes. The pharmacy will create a label: The script will be printed by the pharmacy and include: *Refrigerate * place 3mL of prepared drug into the nebulizer well. Administer twice a day over 10- 15 minutes until vapor is no longer seen, as demonstrated for owner, for 14 days. IL-15 will need to be packaged on ice for transport with care to avoid freeze/thaw.

6.7.4 Return or Destruction of Investigational Product
See Appendix

CONFIDENTIAL
This material is the property of the University of California Do not disclose or use except as authorized in writing by the study sponsor

7 Study Procedures

7.1 Schedule of Events

Procedure	Day -7 to -1	Day 0	Day 7	Day 14	Day 21	Day 35	Day 49	Follow-up q8wks[2]
Caregiver informed consent	X							
MDR Test in susc. breed	X							
Screening echocardiogram	X							
Nebulizer demonstration with saline	X							
Nebulizer sent home for desensitization	X							
Physical Examination	X	X	X	X	X	X	X	X
Quality of Life Form	X	X	X	X	X	X	X	X
CBC	X[1]	X	X	X	X	X		
Serum biochemistry	X[1]		X		X			
Urinalysis	X[1]				X			
PBMCs		X	X	X	X	X		
Thoracic radiographs		X[3]				X	X	X
Doxorubicin administration		X						
IL-15 prepared and sent home with PPE			X					
IL-15 administration (at home)			X[4]	X				

Procedure	Day -7 to -1	Day 0	Day 7	Day 14	Day 21	Day 35	Day 49	Follow-up q8wks[2]
Caregiver informed consent	X							
MDR Test in susc. breed	X							
Screening echocardiogram	X							
Nebulizer demonstration with saline	X							
Nebulizer sent home for desensitization	X							
Physical Examination	X	X	X	X	X	X	X	X
Quality of Life Form	X	X	X	X	X	X	X	X
CBC	X[1]	X	X	X	X	X		
Serum biochemistry	X[1]		X		X			
Urinalysis	X[1]				X			
PBMCs		X	X	X	X	X		
Thoracic radiographs		X (within 1 week)				X	X	X

Doxorubicin administration		X						
IL-15 prepared and sent home with PPE			X					
IL-15 administration (at home)			X	X				

[1]Should be performed within 7 days of Day 0, may occur outside of the UCD-VMTH.

[2]Follow-up visits planned until Day 98, but may continue based on PI and client preference

[3] Should be performed no more than one month before Day 0.

[4] Initial IL-15 treatment will be done in the hospital to monitor patient. All subsequent treatments will be done at home.

7.2 Visit Requirements

7.2.1 Pre-Enrollment Screening – Visit Day -7 to -1

Pre-Enrollment Screening:

- Dogs will be evaluated to determine eligibility by the trials coordinator and trials clinician
- If the dog appears to meet preliminary inclusion criteria, a formal appointment/consultation (trials service) will occur.
- At the initial consult, the owners will be given detailed information on the trial, including nebulization.
- If interested, the owner will review the Informed Consent Form with trials staff and sign the document.
- Quality of life assessment form (baseline)
- Required diagnostics:
    - Cytologic or histologic diagnosis of osteosarcoma or melanoma *(should occur prior to screening visit)*
        - A copy of the histologic or cytologic diagnosis of osteosarcoma or melanoma will be recorded in the study binder and in patient chart.
    - CBC, serum biochemistry, and urinalysis within 7 days of Day 0 *(May occur prior to or after desensitization and outside lab is acceptable)*
    - Screening echocardiogram performed at UC Davis (paid for by the study) within one month of Day 0 (*paid for by study*)
    - MDR mutation status must be determined *for at-risk breeds* prior to Day 0. (*paid for by study*)
    - Clients will be responsible for the cost of obtaining a diagnosis, the initial consultation and required screening diagnostics (CBC, blood chemistry, and urinalysis), as well as any other screening tests deemed appropriate by the attending clinician
- Owners will be trained in nebulization treatments (including a demonstration with the patient) and will be sent home with saline and a nebulizer to desensitize the dog.
- The clinical trial coordinator will document the nebulizer training on the Training Log and the owners will sign the Equipment Agreement.

This material is the property of the University of California Do not disclose or use except as authorized in writing by the study sponsor

- ***Patients requiring > 7 days for desensitization or MDR results remain eligible, however, eligibility/screening criteria will need to adhere to study requirements and therefore may need to be repeated (at the owner's expense) to confirm eligibility.***
- Schedule Day 0 appointment in coordination with medical oncology due to doxorubicin administration and Canter lab for PBMC time points.
- Enrollment – Visit Day 0

7.2.2 Eligibility confirmation and lymphodepletion with doxorubicin- Visit Day 0
- Physical examination and confirmation of eligibility
- Quality of life assessment form
- 11.5 mL blood will be collected for PBMCs and CBC
- Obtain baseline thoracic radiographs (sedation likely required)
- Bloodwork evaluated for adequacy by primary clinician prior to chemotherapy administration
- Doxorubicin @ 30mg/m2 or 1mg/m2 IV depending on clinician prescription, both prepared and administered by medical oncology staff
- Patient sent home under owner's care
- Client provided chemosafety information and routine PRN meds

7.2.3 Follow-up – Visit Day 7

<u>*Start Inhaled IL-15 Therapy:*</u>
- Physical examination
- Quality of life assessment form
- 13 mL of blood obtained for CBC, Chem and PBMC's
- CBC evaluated by trials clinician
    - No minimum cell requirements for IL-15 administration, but if clinically unwell from doxo, may delay
- Inhaled IL-15 (50ug) started in hospital followed by 2 hours monitoring (See XXX section for monitoring details)
- 14 days' supply of IL-15, required PPE, IL-15 Administration Diary sent home with owner

7.2.4 Follow-up – Visit Day 14 *(+/- 3 days)*

<u>*Monitoring and Study Blood:*</u>
- Physical examination
- Quality of life assessment form
- 11.5 mL of blood obtained for CBC and PBMCs

7.2.5 Follow-up – Visit Day 21 *(+/- 3 days)*

<u>*Monitoring, Study Blood & Equipment Return:*</u>
- Physical examination
- Quality of life assessment form
- 13 mL of blood obtained for CBC, Chem and PBMCs
- Urinalysis
- Owner returns nebulizer and unused drug to center
- Owner returns completed Daily Drug Administration Diary

CONFIDENTIAL
This material is the property of the University of California Do not disclose or use except as authorized in writing by the study sponsor

7.2.6 Follow-up – Visit Day 35 *(+/- 3 days)*

Response Evaluation, Bloodwork, & Monitoring:

- Physical examination
- Quality of life assessment form
- 11.5 mL blood will be collected for PBMCs and CBC
- Repeat thoracic radiographs

7.2.7 Follow-up – Visit Day 49 *(+/- 3 days)*

Response Evaluation & Monitoring:

- Physical examination
- Quality of life assessment form
- Repeat thoracic radiographs

7.2.8 Long-term Monitoring *(+/- 3 days)*

Monitoring:

- Physical examination
- Quality of life assessment form
- Repeat thoracic radiographs

7.3 Trial Compliance

7.3.1 Prior and Concomitant Therapy

7.3.1.1 Prior Therapy

- 2 week washout from chemotherapy or corticosteroids
- 4 week washout from radiation therapy and/or immunotherapy
- No prior IL-15 permitted
- Bisphosphonates permitted.
- Prior doxorubicin is permitted
- NSAIDS permitted if patient requires for pain control and received for >2 weeks prior to Day 0
- All medications will be recorded in the study CRF
- Prior therapy will be reviewed during the screening process

7.3.1.2 Concomitant Therapy

Non-study medications/treatments are prohibited during the trial period. Exceptions are as described above (NSAIDS for pain control acceptable if patient has received for > 2 weeks, pamidronate or zoledronate also permitted). Any non-study medications will be captured on the Concomitant Therapy Form.

CONFIDENTIAL
This material is the property of the University of California Do not disclose or use except as authorized in writing by the study sponsor

7.3.1.3 Rescue Therapy

No rescue therapy is available for metastatic osteosarcoma and melanoma, however the patient can be withdrawn from the trial to pursue other therapeutic options.

7.3.2 Lifestyle/Diet Considerations

Normal feeding and housing post procedures until dog is released to their owners. Normal feeding and housing as provided by individual dog owners post visits. Owners will be provided standard chemotherapy safety handling precautions following doxorubicin administration with regard to chemotherapy exposure potential at home. Owners with concerns about chemotherapy (especially those who may be pregnant, nursing, or with children in the home) should consult their physician to see if this trial is appropriate for them. Owners will also be instructed on the protective equipment for handling the IL-15.

7.3.3 Subject Compliance Monitoring

Prior to discharge on Day -7 to-1 (screening) owners will be trained on proper use and handling of nebulizer and drug. Owners will go home with a paper copy of "Owner Administration Instructions for Inhaled IL-15" which includes links to training videos. Proper training will be document on a training log. Owners will complete and return a "Daily Drug Administration Diary" logging Inhaled IL-15 treatments. Failing to comply with treatment regimens and scheduled visits will be grounds for discontinuation in the study.

7.3.4 Protocol Deviations

Any excursions from this protocol will be captured on a Protocol Deviation form and reviewed by the Principal Investigator.

7.4 Clinical Procedures / Assessments

See Visit Descriptions

7.5 Laboratory Procedures / Assessments

See Appendices at the end of the document as well as separate SOP Appendix companion document.

8 Statistical Plan

8.1 Sample Size Determination

Hypothesizing an improvement in ORR from 11% to 50% using conditioning chemotherapy, a sample size of 10 provides 80% power to detect this difference between our historical ORR (the null hypothesis) and the higher ORR of interest at the 0.05 level (1-sided). Therefore, using this study design, it is unlikely that treatments producing modest improvements in ORR will be selected for follow up testing

8.2 Statistical Methods

[Summarize the statistical analysis approach for the trial. Detail the analysis of both primary and secondary endpoints.]

8.3 Subject Population(s) for Analysis

per-protocol/protocol compliant (starting inhaled IL-15)

9 Safety and Adverse Events

9.1 Definitions

Adverse Event (AE)
An ***adverse event*** (AE) is any symptom, sign, illness or experience that develops or worsens in severity during the course of the study. Intercurrent illnesses or injuries should be regarded as adverse events. Abnormal results of diagnostic procedures are considered to be adverse events if the abnormality:

- results in study withdrawal
- is associated with a serious adverse event
- is associated with clinical signs or symptoms
- leads to additional treatment or to further diagnostic tests
- is considered by the investigator to be of clinical significance

Serious Adverse Event (SAE)
Adverse events are classified as serious or non-serious. A ***serious adverse event*** (SAE) is any AE that is:

- fatal
- life-threatening
- requires or prolongs hospital stay
- results in persistent or significant disability or incapacity
- an important medical event

Important medical events are those that may not be immediately life threatening, but are clearly of major clinical significance. They may jeopardize the pet and may require intervention to prevent one of the other serious outcomes noted above. All adverse events that do not meet any of the criteria for serious should be regarded as ***non-serious adverse events***.

Adverse Event Reporting Period
Adverse events that occur from the time of screening to study completion are recorded.

Preexisting Condition
A preexisting condition (one that is present at the start of the study) is recorded as an adverse event if the frequency, intensity, or the character of the condition worsens during the study period.

General Physical Examination Findings
At screening, any clinically significant abnormality is recorded as a preexisting condition. Any new clinically significant findings/abnormalities that meet the definition of an adverse event are recorded and documented as an adverse event during the trial.

CONFIDENTIAL
This material is the property of the University of California Do not disclose or use except as authorized in writing by the study sponsor

Laboratory Value Findings
CBC, chemistry, and urinalysis values outside reference ranges at the follow-up visit or at unscheduled interim visits will be recorded as adverse events.

Owner Reported Findings
Quality of Life forms will be monitored and any reported changes in health status from baseline will be recorded as an adverse event.

Post-study Adverse Event
All unresolved adverse events are followed by the investigator until the events are resolved, the subject is lost to follow-up, or the adverse event is otherwise explained. At the last scheduled visit, the investigator instructs each caregiver to report any subsequent event(s) that they, or the pet's primary veterinarian, believes might reasonably be related to participation in this study. The investigator notifies Sponsor of any death or adverse event occurring at any time after a subject has discontinued or terminated study participation that may reasonably be related to this study.

9.2 Recording of Adverse Events

At each contact with the caregiver, the investigator or research assistant seeks information on adverse events by specific questioning. Caregivers will be asked to fill out a Quality of Life form to monitor patient health.

Information on all adverse events is recorded immediately in the source document, and also in the appropriate adverse event module of the case report form (CRF and REDCap). All clearly related signs, symptoms, and abnormal diagnostic procedures results are recorded in the source document.

All adverse events occurring during the study period are recorded. The clinical course of each event is followed until resolution, stabilization, or until it is determined that the study treatment or participation is not the cause. Serious adverse events that are still ongoing at the end of the study period are followed up to determine the final outcome. Any serious adverse event that occurs after the study period and is considered to be possibly related to the study treatment or study participation is recorded and reported immediately.

9.3 Reporting of Serious Adverse Events and Unanticipated Problems.

9.3.1 Investigator reporting: notifying the study sponsor

A serious adverse event is reported to the study sponsor by telephone within 24 hours of the event. A Serious Adverse Event (SAE) form is completed by the investigator and faxed within 24 hours. The investigator keeps a copy of this SAE form on file at the study site. Report serious adverse events by phone to: Rob Rebhun

At the time of the initial report, the following information is provided:

CONFIDENTIAL
This material is the property of the University of California Do not disclose or use except as authorized in writing by the study sponsor

- Study identifier
- Study Center
- Subject number
- A description of the event
- Date of onset
- Current status
- Whether study treatment was discontinued
- The reason why the event is classified as serious
- Investigator assessment of the association between the event and study treatment

Within the following 48 hours, the investigator provides further information on the serious adverse event in the form of a written narrative. This includes a copy of the completed Serious Adverse Event form, and any other diagnostic information that assists the understanding of the event.

9.4 Stopping Rules

Study participant can be discontinued from trial participation:

- If deemed clinically indicated by the attending clinician
- If requested by the owner
- Owner non-compliance with treatments or visits

9.5 Medical Monitoring

It is the responsibility of the Principal Investigator to oversee the safety of the study. This safety monitoring will include careful assessment and appropriate reporting of adverse events as noted above. Medical monitoring will include a regular assessment of the number and type of serious adverse events.

10 Data Handling and Record Keeping

10.1 Confidentiality

Information about participating pets and their caregivers is kept confidential. The Health Insurance Portability and Accountability Act of 1996 (HIPAA) does not apply to the caregiver as no health care service (diagnosis or treatment) is being provided. No such protected health information (PHI) regulations currently exist for companion animals. Confidentiality of information is described in the informed consent that the caregiver signs prior to enrolling their pet in the study. In the event a caregiver withdraws informed consent, the investigator retains the ability to use all information collected prior to the revocation of consent.

10.2 Source Documents

Source data is all information, original records of clinical findings, observations, or other activities in a clinical trial necessary for the reconstruction and evaluation of the trial. Source data are contained in source documents. Examples of these original documents, and data records include: hospital records, clinical and office charts, laboratory notes, memoranda, subjects' diaries or evaluation checklists, pharmacy dispensing records, recorded data from automated instruments, copies or transcriptions certified after verification as being accurate and complete, microfiches,

photographic negatives, microfilm or magnetic media, x-rays, subject files, and records kept at the pharmacy, at the laboratories, and at medico-technical departments involved in the clinical trial.

10.3 Participant Binder

There will be one comprehensive subject binder to contain all paperwork for the visits for each dog, as well as the study protocol. The binder will contain the following forms:

- Study Requirements Checklist
- Patient Demographic and History
- Signed and dated Owner Informed Consent
- Informed Consent Process Documentation
- Baseline Medical History
- Inclusion/Exclusion Criteria Form
- Clinical Visit Form for each visit
- Quality of Life Form for each visit
- Owner Equipment Agreement
- Initial Inhaled IL-15 Administration and Monitoring Form
- Daily Drug Administration Diary
- Protocol Deviation Form
- Subject Final Status Form

10.4 Case Report Forms

The study case report form (CRF) is the primary data collection instrument for the study. All Case Report Forms much include a subject ID on each page. All data requested on the CRF are recorded. All missing data are explained. If a space on the CRF is left blank because the procedure was not done or the question was not asked, “N/D” is written. If the item is not applicable to the individual case, “N/A” is written. All entries are printed legibly in black ink. If any entry error is made, to correct such an error, a single straight line is drawn through the incorrect entry and the correct data entered above it. All such changes are initialed and dated. Errors are not erased or whited out. For clarification of illegible or uncertain entries, the clarification is printed above the item, then initialed and dated.

10.5 Electronic Data Capture

Records will be manually entered into a REDCap database. The UCD REDCap will be used to electronically collect QOL surveys from pet owners. Adverse events, protocol deviations, Concomitant Medications, IL-15 and doxorubicin administration will also be databased in REDCap. Basic patient and owner information (ID numbers, e-mails, etc.) will be included to facilitate submission of surveys and collection of AEs.

10.6 Records Retention

It is the investigator’s responsibility to retain study essential documents for at least 5 years after initial publication of study results. All paper source documents and CRFs will be stored in individual subject-specific binders maintained on-site at the study center in a climate-controlled pass-key protected room. Each paper form will also be scanned into digital form and saved in individual subject-specific folders in encrypted form on an internet-based server that is password-

CONFIDENTIAL
This material is the property of the University of California Do not disclose or use except as authorized in writing by the study sponsor

protected by a two-step verification process, and a password-protected secure university network server that is backed up nightly.

11 Study Monitoring, Auditing, and Inspecting

11.1 Study Monitoring

Data will be entered by the trials coordinator and monitoring provided by the study PI.

11.2 Auditing and Inspecting

The investigator will permit study-related monitoring, audits, and inspections by the IACUC/ CTRB, the sponsor, government regulatory bodies, and University compliance and quality assurance groups of all study related documents (e.g. source documents, regulatory documents, data collection instruments, study data etc.). The investigator will ensure the capability for inspections of applicable study-related facilities (e.g. pharmacy, diagnostic laboratory, etc.).
Participation as an investigator in this study implies acceptance of potential inspection by government regulatory authorities and applicable University compliance and quality assurance offices.

12 Ethical Considerations

This study is to be conducted according to applicable government regulations and Institutional research policies and procedures. While the US and international standards of Good Clinical Practice (FDA Title 21 part 312 and International Conference on Harmonization guidelines) apply to human research trials, much of the guidance is applicable to companion animal trials and will be conducted accordingly.

This protocol and any amendments will be submitted to a properly constituted Institutional Animal Care and Use Committee or Institutional Review Board (IRB), in agreement with local legal prescriptions, for formal approval of the study conduct. The decision of the IACUC/CTRB concerning the conduct of the study will be made in writing to the investigator and a copy of this decision will be provided to the sponsor before commencement of this study.

All caregivers for this study are provided a consent form describing this study and providing sufficient information for caregivers to make an informed decision about their own and their pet's participation in this study. See Attachments for a copy of the Owner Informed Consent Form. These consent forms will be submitted with the protocol for review and approval by the CTRB and IACUC for the study. The formal consent of a caregiver, using the CTRB and IACUC-approved consent forms, must be obtained before the animal is submitted to any study procedure. These consent forms must be signed by the caregiver and the investigator-designated research professional obtaining the consent.

13 Study Completion and Closeout Procedures

Once the trial is complete, the coordinator will complete an End of Trial Review form for each subject to double check that all necessary source data is present in the file and that all required data

CONFIDENTIAL
This material is the property of the University of California Do not disclose or use except as authorized in writing by the study sponsor

has been entered into EDC. Subject source binders will be returned to the Investigator or housed in the VCCT archives, per the Investigator's discretion.

14 Study Finances

14.1 Funding Source

This study is funded by the NIH

14.2 Conflicts of Interest

Any investigator who has a conflict of interest with this study (patent ownership, royalties, or financial gain greater than the minimum allowable by their institution, etc.) will have the conflict reviewed by a properly constituted Conflict of Interest Committee with a Committee-sanctioned conflict management plan that has been reviewed and approved by the study sponsor prior to participation in this study. All University of California investigators will follow the University conflict of interest policy.

14.3 Subject Stipends or Payments

Caregivers will not receive any study stipends or payments.

Costs Covered by Study:
Once determined eligible and the pet is enrolled, the study will cover the cost of appointment fees, blood sampling, sedation, thoracic radiographs, single dose of doxorubicin + administration, and study related IL-15, PPE, and procedures. The study will provide the use of the nebulizer, however, this will need to be returned at the end of the treatment. Study will also cover costs of screening echocardiogram and MDR testing in at-risk breeds.

Costs NOT Covered by Study:
Owners are responsible for eligibility/screening which includes initial or recheck office examination, bloodwork including CBC/Chemistry panel, urinalysis, thoracic radiographs, and confirmed diagnosis of osteosarcoma or melanoma. Additional diagnostics may be recommended based on clinician preference and would be at the owner's expense.

If the pet experiences an adverse event(s) as a result of taking part in this study, and is in need of medical treatment, the study sponsors will offer to pay for medical treatment for injury/side effects up to $2000.The study can ONLY pay for costs of therapy incurred at the UC Davis Veterinary Medical Teaching Hospital. Costs associated with treatment beyond $2000 will be at the expense of the owner.

15 Publication Plan

Neither the complete nor any part of the results of the study carried out under this protocol, nor any of the information provided by the sponsor for the purposes of performing the study, will be published or passed on to any third party without the consent of the principal investigator. Any investigator or research assistant involved with this study is obligated to provide the principal investigator with complete test results and all data derived from the study.

16 Veterinary Center for Clinical Trials Standard Operating Procedures

This study is subject to the following VCCT SOPs, which will be reviewed with study personnel involved in relevant study procedures and logged per VCCT policy.

16.1 Standard Operating Procedures for Doxorubicin

16.2 Oncology Antineoplastic Hazardous Drug Handling Policy

17 References

[Provide the citations for all publications and presentations referenced in the text of this document.]

18 Attachments

18.1 Appendix I

18.2 Sample Collection & Processing

18.2.1 Sample collection and processing (<u>Day -7</u> Pre-Enrollment):

18.2.1.1 Collect 6-8mL blood for CBC/Chem (+/- MDR mutation testing if at risk breed*), ≥3mL urine for standard UA

18.2.1.1.1 Process as usual per VMTH protocol

18.2.1.1.1.1 *Submit MDR mutation test with Research Sample Request form if performed

18.2.1.2 Place torn patient sticker on window portion of <u>all</u> sample tubes. Do not cover tube sticker or write patient info on it.

18.2.2 Sample collection and processing (<u>Day 0</u> Enrollment & Lymphodepletion):

18.2.2.1 Collect 10mL of whole blood in (2) 5mL (K3) EDTA tube for PBMCs

18.2.2.1.1 Sample will be refrigerated until pick-up

18.2.2.1.2 See contact information (Canter lab) below for same-day pick-up. Canter lab should be notified a minimum of 24-hours in advance

18.2.2.2 Collect 6mL blood for CBC/Chem

18.2.2.2.1 Process as usual per VMTH protocol and submit with Research Sample Request form.

18.2.2.3 Place torn patient sticker on window portion of <u>all</u> sample tubes. Do not cover tube sticker or write patient info on it.

18.2.2.3.1 Submit cytology per VMTH protocol with Research Sample Request form

CONFIDENTIAL
This material is the property of the University of California Do not disclose or use except as authorized in writing by the study sponsor

18.2.3 Sample collection and processing (Days 7, 14, and 35):

18.2.3.1 Collect 10mL of whole blood in (2) 5mL (K3) EDTA tube for PBMCs

18.2.3.1.1 Sample will be refrigerated until pick-up

18.2.3.1.2 See contact information (Canter lab) below for same-day pick-up. Canter lab should be notified a minimum of 24-hours in advance

18.2.3.2 Collect 3mL blood for CBC

18.2.3.2.1 Process as usual per VMTH protocol and submit with Research Sample Request form.

18.2.3.3 Place torn patient sticker on window portion of all sample tubes. Do not cover tube sticker or write patient info on it.

18.2.4 Sample collection and processing (Day 21 Follow-Up):

18.2.4.1 Collect 10mL of whole blood in (2) 5mL (K3) EDTA tube for PBMCs

18.2.4.1.1 Sample will be refrigerated until pick-up

18.2.4.1.2 See contact information (Canter lab) below for same-day pick-up. Canter lab should be notified a minimum of 24-hours in advance

18.2.4.2 Collect 6mL blood for CBC/Chem and ≥3mL urine for standard UA

18.2.4.2.1 Process as usual per VMTH protocol and submit with Research Sample Request form.

18.2.4.3 Place torn patient sticker on window portion of all sample tubes. Do not cover tube sticker or write patient info on it.

18.2.5 For Pick-up:

[redacted]

[redacted]

[redacted]

Appendix II

SOP07-A Drug Preparation, Handling, and Dispensation of inhaled rhIL-15

1.0 Purpose:

The purpose of this standard operating procedure (SOP) is to explain the proper procedures, handling, and dispensation of IL-15

2.0 Scope/Responsibility:

These procedures are to be followed by staff responsible for requesting, receiving, and dispensation for client to use the IL-15.

3.0 Request, Shipping, Receipt, and Dispensing:

Request for rhIL-15:

- Sites will be responsible for requesting rhIL-15 shipment from UC Davis.
- To facilitate timely shipment, please email Robert Rebhun [redacted] and Daniel York [redacted] as soon as the surgery date is determined.
- Upon receipt of patient information and anticipated surgery date, UC Davis will notify coordinators/site PI of the planned shipment date.

Receipt and dispensation of rhIL-15 for nebulization:

- Upon receipt, site is to immediately place IL-15 at 4C.
- Inspection of syringes and Temperature Tracking Device/Card – Please confirm that proper temperature was maintained during shipping and transport. If temperature tracking device is acceptable, please obtain and email a photo of the temperature tracking device to Robert Rebhun [redacted] and Daniel York [redacted] If concerns, please also send an image and contact Dr. Rebhun or Dr. York immediately.
- Each site pharmacy will create a label: The script will be printed by the pharmacy and include: ***Refrigerate *** place 3mL of prepared drug into the nebulizer well. Administer twice a day over 10-15 minutes or until vapor is no longer seen, as demonstrated for owner, for 14 days.

7.0 Reconciliation of study drug:

IL-15 vials will be recorded on the drug accountability log and recorded dose per patient. This will account for the drug used and the drug left over at the end of the study.

8.0 Destruction of Study Drug:

Any unused drug will be returned to the participating site and will be held at 4C until the patient is removed from study, at which time unused drug will be returned to UC Davis.

CONFIDENTIAL
This material is the property of the University of California Do not disclose or use except as authorized in writing by the study sponsor

IL-15 Drug Preparation, Handling, and Dispensation of inhaled rhIL-15

1.0 Purpose:

The purpose of this standard operating procedure (SOP) is to explain the proper procedures, preparation and dispensation of IL-15

2.0 Scope/Responsibility:

These procedures are to be followed by staff responsible for reconstituting, drawing up and packaging for client to use the IL-15.

3.0 Materials:

- 3x 18g x 1 ½ needle (or similar)
- 1x 10 or 20mL syringe (to remove saline)
- 1x 1mL syringe (to add BSA)
- 1x 3mL syringe (to add IL-15)
- rhIL-15 is stored in -80C freezer
- 9% BSA is stored at -20C freezer (PROVIDED BY UC DAVIS)
- 100mL 0.9% saline bag ***stored at 4C***

Volume chart for making up 90mL of IL-15 final working concentrations with 0.1% BSA and 0.9% Saline. rhIL-15 stock = 510ug/mL in solution of 25mM Sodium Phosphate, 500mM Sodium Chloride, pH 7.4. 1mL volume per vial. Stored at -80C

Actual Dose	Actual IL15 Conc.	Saline Vol. to Discard	9% BSA to Add	Stock IL-15 (510μg/mL) to Add	Vials of IL-15 to Thaw
50ug	16.7 μg/mL	14.0 mL	1.0 mL	3.0 mL	3

4.0 Procedure:

IL15 Formulation:

1. Place 100mL 0.9% saline bag at 4C (if not already)
2. Thaw 1x vial of 9% BSA at room temp
3. Thaw 3 vials of rhIL-15 (1mL of 510μg/mL per vial)
4. Remove 14.0 mL of saline from 100mL saline bag
5. Add 1.0mL of 9% BSA to the saline bag and mix well
   *****NOTE: Add BSA and mix BEFORE adding IL-15 to saline bag *****
6. Add 3.0mL of rhIL-15 stock to saline bag and mix well
7. Fill 30 x 5mL syringes with **exactly 3mL** of IL-15 solution in each.
8. Cap syringes with needleless syringe caps and **store refrigerated at 4C**
9. Store at 4C until shipment, DO NOT FREEZE

18.2.5.4

Appendix III
<u>Owner Administration Instructions for inhaled rhIL-15</u>

Desensitization and Positive-Reward Training for Inhaled IL-15 Therapy

It is important to introduce this therapy in a stepwise approach, adding only one "new" thing at a time. Desensitization moves at a different pace for each dog, but it is critical that we match their pace so that we don't accidentally enforce avoidance behavior. This being said, nebulization is a lot to ask, and some dogs may simply be too anxious to accept this treatment. In addition, if the nosecone is not working, we can try an alternative "bag" approach which is potentially less confining. Ultimately our goal is to train them to rest comfortably and undergo nebulization therapy for around 10-12 minutes twice daily. You obviously know your dog best, but we think the following outlined steps should be useful as you move through this training.

1. Using the cone or mask by itself (no tubing etc.), place the cone over the muzzle for just a second or two followed by a reward treat. Do this several times over the course of the first day gradually increasing the time as tolerated. Avoid having your pet struggle, simply build up the time based on their comfort and always follow with a reward treat and positive reassurance.
2. Find a designated, well ventilated, and cool area within your home that has a power outlet where treatment sessions can take place. Have your pet sit or lay with you in this spot and continue to introduce the cone for increasing periods of time, followed by treat rewards. Of course, belly petting, reassurance and comforting is always encouraged!
3. Now, go ahead and connect the tubing to the nosecone (not attached to the nebulizer), just so they get used to this addition, and continue to practice for longer periods of time.
4. Once your dog is comfortable with the cone and the space, it is time to introduce the nebulizer. This should also be introduced stepwise. First plug the nebulizer in, keep it turned off, but keep it next to you while you are continuing to desensitize to the cone. Once you feel your pet is ready, you can turn the nebulizer on and let it run (so they get used to the noise)
5. The next step is to actually connect the nebulizer to the tubing and cone. This will introduce a stream of air into the nosecone, which will be new for them. Again, move at their pace and continue with positive reinforcement.
6. Once they are comfortable with the cone, the noise, and the airflow; you should add 3 milliliters of saline to the nebulizer cup. This will create the vapor which they will recognize as new but should get used to fairly quickly.
7. Last but not least, during these sessions, your dog will need to get used to your personal protective equipment (PPE)! This will include your gown, gloves, eye protection, and mask. Remember, we just want to introduce one of each of these new things at a time. We would suggest you move in the order of gown, gloves, goggles, and then mask. It may be helpful to have your dog see you put these on.
8. Once your dog has received the 14 days of treatment please remember to collect all the Personal protective equipment, the nebulizer, hose and mask, and any unused drug and supplies and return it to UC Davis at your appointment the following day.
9. **<u>Training video specific for this study:</u>**

This material is the property of the University of California Do not disclose or use except as authorized in writing by the study sponsor

   a. https://ucdavis.box.com/s/oqpdqj9vo5ew4adyyw279u2zhj71oyil

10. You Tube videos if you have any questions regarding your machine:
    a. https://www.youtube.com/watch?v=d5AASiAdLiU
    b. https://www.youtube.com/watch?v=rJitnKowVBg
    c. https://www.youtube.com/watch?v=xbj4z4-R7eE

Equipment Checklist:

- ☐ Nebulizer
- ☐ Face cone/clear boot (x2)
- ☐ 1 Nebulizer Set
- ☐ Labeled Drug for study

Personal Protective Equipment:

- ☐ Two splash resistant protective disposable gowns
- ☐ Goggles
- ☐ n95 mask (x2)
- ☐ Supply of gloves (30 pair)

18.2.5.5

CONFIDENTIAL
This material is the property of the University of California Do not disclose or use except as authorized in writing by the study sponsor
